# Amylimycins A–C, New Bacillomycin D Analogs from Marine-Derived *Bacillus amyloliquefaciens*

**DOI:** 10.3390/md24060218

**Published:** 2026-06-17

**Authors:** Jaeyoun Lee, Seung Hyun Kim, Soohyun Um

**Affiliations:** 1 College of Pharmacy, Yonsei Institute of Pharmaceutical Sciences, Yonsei University, Incheon 21983, Republic of Korea; jaeyoun1024@yonsei.ac.kr; 2 Department of Forest Products and Biotechnology, Kookmin University, Seoul 02707, Republic of Korea

**Keywords:** lipopeptides, bacillomycin D, *Bacillus amyloliquefaciens*, antibacterial activities

## Abstract

Marine-derived microorganisms are a rich source of structurally diverse natural products with significant pharmaceutical potential. In this study, three new cyclic lipopeptides, amylimycins A–C (**1**–**3**), were isolated from a marine-derived *Bacillus amyloliquefaciens* strain. The chemical structures of these compounds were elucidated through comprehensive spectroscopic analyses and chiral derivatization using 1-fluoro-2,4-dinitrophenyl-5-alanine amide (FDAA). Amylimycins A–C (**1**–**3**) were identified as bacillomycin D analogs belonging to the iturin family, characterized by a cyclic heptapeptide core linked to a β-amino fatty acid moiety. Notably, these compounds featured uncommon branched β-amino fatty acid chains with varied chain lengths, representing a distinctive structural characteristic among bacillomycin D analogs. Amylimycins A–C (**1**–**3**) showed moderate antibacterial activity against the Gram-positive bacteria *Bacillus subtilis* and *Staphylococcus epidermidis*, while displaying weak to no activity against the Gram-negative strains *Escherichia coli* and *Pseudomonas fluorescens*.

## 1. Introduction

Marine microorganisms have emerged as a rich source of structurally diverse and biologically active natural products, many of which have exhibited significant pharmaceutical potential [1,2]. Among these, lipopeptides produced by marine-derived bacteria have attracted considerable attention because of their potent antibacterial, antifungal, and antiviral activities [3,4,5]. Lipopeptides are amphiphilic molecules consisting of a lipid moiety linked to a peptide structure, typically comprising a hydrophobic fatty acid tail and a hydrophilic peptide ring or chain [6]. This amphiphilic structural feature enables them to function as effective biosurfactants and to strongly interact with biological membranes [7]. Lipopeptides are produced by various microorganisms, including bacteria, fungi, and yeast. Among these, lipopeptides derived from *Bacillus* species have been most extensively studied [8]. Representative groups include surfactins, fengycins, and iturins; each is characterized by distinct peptide compositions, amino acid sequences, fatty acid chain lengths, and fatty acid moieties [9].

From a biological standpoint, lipopeptides primarily exert their antimicrobial effects by disrupting microbial membranes [10]. Several members of this class have been successfully developed into clinically relevant therapeutics. For example, daptomycin, a cyclic lipopeptide antibiotic, has become a key therapeutic agent for the treatment of severe infections caused by Gram-positive bacteria, particularly methicillin-resistant *Staphylococcus aureus* (MRSA) [11]. Similarly, echinocandins, such as caspofungin, represent antifungal lipopeptides that inhibit β-glucan biosynthesis in fungal cell walls and are particularly effective against *Candida* species [12]. Moreover, pumilacidins derived from *B. pumilus* have exhibited antiviral activity against herpes simplex virus, along with other therapeutic effects [13].

**Figure 1:**
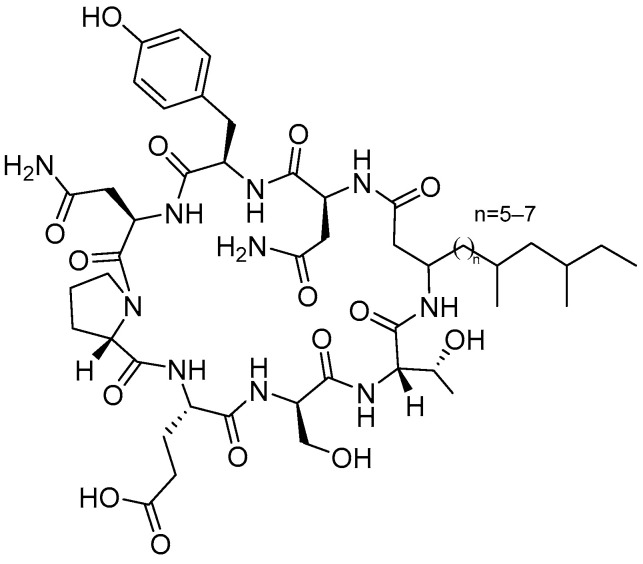
Chemical structures of amylimycins A–C (**1**–**3**).

Within this context, *B. amyloliquefaciens* is recognized as a prolific producer of cyclic lipopeptides, particularly surfactins and bacillomycin D analogs [14]. Bacillomycin D belongs to the iturin family of lipopeptides and is characterized by a cyclic heptapeptide linked to a β-amino fatty acid chain. This structural framework confers strong amphiphilicity, facilitating efficient interaction with biological membranes. Bacillomycin D and its analogs are well known for their potent antifungal activity, primarily through the disruption of membrane integrity via interactions with sterols, resulting in increased membrane permeability and cell lysis [15]. In addition to antifungal effects, some bacillomycin D derivatives have been reported to exhibit antibacterial and antiviral effects, highlighting their broad-spectrum bioactivity [14,16]. In this study, we report the isolation and structural elucidation of new bacillomycin D analogs, amylimycins A–C, obtained from a marine-derived *B*. *amyloliquefaciens* strain (Figure 1). We determined their structures using comprehensive spectroscopic analyses, including high-resolution mass spectrometry (HRMS) and nuclear magnetic resonance (NMR) techniques. We also determined the absolute configurations of the amino acid residues in the amylimycins through chiral derivatization. Furthermore, we evaluated their antibacterial activities against both Gram-positive and Gram-negative bacteria, demonstrating their potential as novel antimicrobial agents.

## 2. Results and Discussion

**Figure 2:**
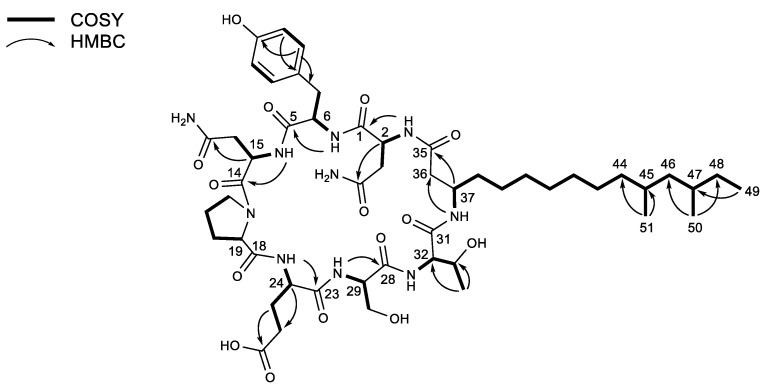
Key COSY and HMBCs of **1**.

Amylimycin A (**1**) was purified as a white amorphous powder. Its molecular formula was determined to be C_51_H_80_N_10_O_15_, with an exact mass of *m*/*z* 1073.5885 [M + H]^+^ (calculated for C_51_H_81_N_10_O_15_, *m*/*z* 1073.5877), as confirmed by HRMS. The structure was determined using a combination of 1D and 2D NMR methods, including heteronuclear single quantum coherence (HSQC), correlation spectroscopy (COSY), heteronuclear multiple bond correlation (HMBC), and rotating-frame Overhauser effect spectroscopy (ROESY), together with infrared (IR) spectroscopy and mass spectrometry (MS). The NMR data for amylimycin A revealed the presence of 11 carbonyls (δ_C_ 174.7, 173.3, 172.1, 171.5, 171.4, 170.4, 170.3, 169.9, 169.6, 169.2, and 169.0) and seven amide protons (δ_H_ 8.54, 8.42, 8.32, 8.06, 8.04, 7.58, and 7.56), suggesting the presence of a peptide. Moreover, the 1D and 2D NMR (HSQC, COSY, HMBC, ROESY, and TOCSY) data for amylimycin A revealed the amino acid composition, which included two asparagine (Asn) residues, along with tyrosine (Tyr), proline (Pro), glutamic acid (Glu), serine (Ser), and threonine (Thr). The sequence of the seven amino acid residues was determined based on HMBC signals and ROESY correlations. Key HMBCs were observed: 6-NH of Tyr (δ_H_ 7.56) to C-1 of Asn-1 (δ_C_ 170.3), 15-NH of Asn-2 (δ_H_ 8.54) to C-5 of Tyr (δ_C_ 170.4), 24-NH of Glu (δ_H_ 8.04) to C-18 of Pro (δ_C_ 169.0), 29-NH of Ser (δ_H_ 8.42) to C-23 of Glu (δ_C_ 171.5), and 32-NH of Thr (δ_H_ 8.06) to C-28 of Ser (δ_C_ 173.3). Moreover, the HMBC signals from 2-NH of Asn-1 (δ_H_ 8.32) to C-35 of a β-amino acid (δ_C_ 169.9) and those from 37-NH of the β-amino acid (δ_H_ 7.58) to C-31 of Thr (δ_C_ 169.6) established the linkage between Asn-1 and the β-amino acid and between the β-amino acid and Thr, respectively (Figure 2). The arrangement of amino acid residues was also confirmed through ROESY correlations between the amide NH and the α proton of the adjacent amino acid. Consequently, the planar structure of amylimycin A was determined to be a cyclic lipopeptide similar to bacillomycin D. The HMBC data of the β-amino acid in amylimycin A revealed signals from CH_3_-51 (δ_H_ 0.84) to C-45 (δ_C_ 27.4), from CH_3_-50 (δ_H_ 0.81) to C-47 (δ_C_ 33.8), and from CH_3_-49 (δ_H_ 0.82) to C-47 (δ_C_ 33.8) and C-48 (δ_C_ 29.0), indicating that the β-amino acid is a newly characterized 3-amino-12,14-dimethylpentadecanoic acid. Consequently, the planar structure of compound **1** was determined to be cyclo[Asn-1-Tyr-Asn-2-Pro-Glu-Ser-Thr-3-amino-12,14-dimethylpentadecanoic acid] (Figures S1–S7, Table 1 and Table 2).

To determine the absolute configuration of amino acids in **1**, l- and d-FDAA (1-fluoro-2,4-dinitrophenyl-5-l-/d-alanine amide) derivatization was performed. The retention times of the l- and d-FDAA-derivatized hydrolysates were 26.8 and 24.1 min for Tyr, 8.9 and 10.2 min for Pro, 7.1 and 7.8 min for Glu, 5.8 and 5.1 min for Ser, and 5.3 and 8.0 min for Thr, respectively. Two Asn-derived peaks were observed at 12.6 and 13.5 min, indicating the presence of both l- and d-Asn residues. This assignment is supported by the previously reported domain organization of the bacillomycin D biosynthetic gene cluster [17]. In the biosynthetic assembly line, the module responsible for incorporation of Asn-1 lacks an epimerization (E) domain and therefore is expected to yield an l-configured residue, whereas the module incorporating Asn-2 contains an E domain that converts the initially activated l-Asn to d-Asn during peptide assembly [17]. Thus, Asn-1, Pro, Glu, and Thr in amylimycin A (**1**) were determined to be l-amino acids, whereas Tyr, Asn-2, and Ser were determined to be d-amino acids (Figures S24 and S25). Previous studies have assigned the β-amino fatty acid stereocenter of bacillomycin D homologs as 3*R* [18]. However, the absolute configuration at C-37 was not directly determined in the present study and therefore remains undetermined for compounds **1**–**3**.

Amylimycin B (**2**) was purified as a white amorphous powder. Its molecular formula was determined to be C_50_H_78_N_10_O_15_, with an exact mass of *m*/*z* 1059.5681 [M + H]^+^ (calculated for C_50_H_79_N_10_O_15_, *m*/*z* 1059.5720). The ^1^H and ^13^C NMR spectra of compound **2** closely resembled those of compound **1**, with the only difference being the absence of a methylene unit in the β-amino acid, as confirmed by the analysis of COSY, TOCSY, HSQC, and HMBC spectra (Figures S8–S15). The HMBCs of the β-amino acid in amylimycin B revealed cross-peaks from CH_3_-50 (δ_H_ 0.84) to C-44 (δ_C_ 27.3), from CH_3_-49 (δ_H_ 0.81) to C-46 (δ_C_ 33.4), and from CH_3_-48 (δ_H_ 0.82) to both C-46 (δ_C_ 33.4) and C-47 (δ_C_ 29.1). These data indicate that the β-amino acid moiety is a novel β-amino acid, identified as 3-amino-11,13-dimethyltetradecanoic acid. Therefore, the planar structure of compound **2** was established as cyclo[Asn-1-Tyr-Asn-2-Pro-Glu-Ser-Thr-3-amino-11,13-dimethyltetradecanoic acid] (Table 1 and Table 2).

Amylimycin C (**3**) was obtained as a white amorphous powder. Its molecular formula was determined to be C_49_H_76_N_10_O_15_, with an exact mass of *m*/*z* 1045.5538 [M + H]^+^ (calculated for C_49_H_77_N_10_O_15_, *m*/*z* 1045.5564). The ^1^H and ^13^C NMR spectra of compound **3** were highly similar to those of compounds **1** and **2**, with the only difference being the loss of methylene units in the β-amino acid moiety, which was verified based on 1D and 2D NMR spectra (Figures S16–S22). The HMBCs observed for the β-amino acid residue in **3** included cross-peaks between CH_3_-49 (δ_H_ 0.83) and C-43 (δ_C_ 27.4), CH_3_-48 (δ_H_ 0.81) and C-45 (δ_C_ 32.9), as well as CH_3_-47 (δ_H_ 0.82) and both C-45 (δ_C_ 32.9) and C-46 (δ_C_ 29.1). Based on these correlations, the β-amino acid unit was identified as 3-amino-10,12-dimethyltridecanoic acid. Accordingly, the planar structure of compound **3** was determined to be cyclo[Asn-1-Tyr-Asn-2-Pro-Glu-Ser-Thr-3-amino-10,12-dimethyltridecanoic acid] (Table 1 and Table 2).

**Figure 3:**
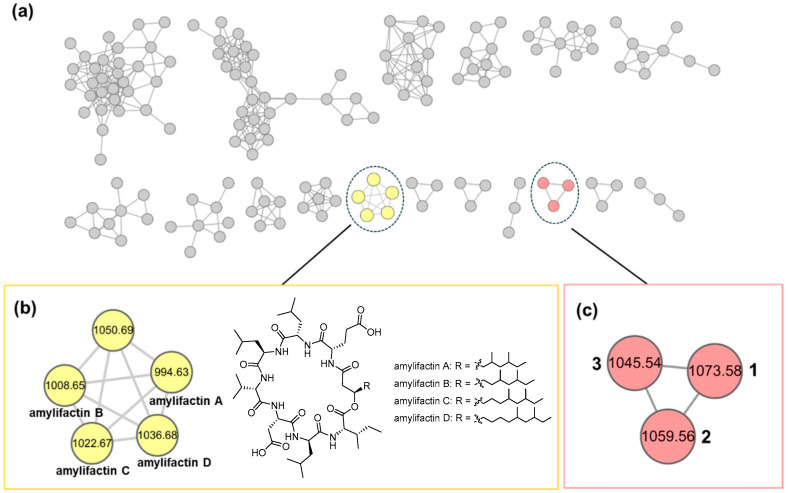
(**a**) GNPS cluster assigned to lipopeptides from the ethyl acetate extract of *B*. *amyloliquefaciens*. (**b**) Cluster corresponding to the known amylifactins A–D and their associated molecular features. (**c**) Target molecular network cluster containing the newly identified lipopeptides. Numbers on nodes denote compounds **1**–**3**. **1**, amylimycin A (*m*/*z* 1073.58 [M + H]^+^); **2**, amylimycin B (*m*/*z* 1059.56 [M + H]^+^); and **3**, amylimycin C (*m*/*z* 1045.54 [M + H]^+^).

Molecular networking analysis of the ethyl acetate extract of *B*. *amyloliquefaciens* revealed that the lipopeptides were organized into a distinct molecular cluster containing amylimycins A–C (**1**–**3**). This clustering pattern indicates a high degree of similarity in their MS/MS fragmentation profiles, suggesting that these compounds share closely related structural features. In particular, the grouping of amylimycins within the same cluster suggests a common biosynthetic origin and comparable core scaffolds with minor structural variations. These molecular networking data provide supporting evidence for the structural relatedness of these compounds and facilitate the identification of new analogs within the same chemical family (Figure 3a). In addition, a separate cluster corresponding to the previously reported amylifactins A–D was also observed [19], supporting the reliability of the molecular networking analysis for dereplication of known metabolites (Figure 3b). Furthermore, a new target cluster composed of unidentified lipopeptide-related nodes was detected, suggesting the presence of additional analogs within this molecular family (Figure 3c).

To evaluate the antibacterial activity of amylimycins A–C (**1**–**3**) against Gram-positive bacteria, including *Bacillus subtilis* and *Staphylococcus epidermidis*, and Gram-negative bacteria, including *Escherichia coli* and *Pseudomonas fluorescens*, a minimum inhibitory concentration (MIC) assay was conducted. Amylimycins A–C (**1**–**3**) were serially diluted in twofold dilutions, with concentrations ranging from 128 to 0.25 µg/mL, to evaluate their antibacterial activity. The antibacterial activities of **1**–**3** were more pronounced against Gram-positive bacteria than against Gram-negative bacteria. In particular, **1**–**3** exhibited moderate activity against *B*. *subtilis* and *S*. *epidermidis*, whereas weak or no activity was observed against *E*. *coli* and *P*. *fluorescens*. This selective activity may be associated with differences in the cell envelope structures of Gram-positive and Gram-negative bacteria, particularly the presence of an outer membrane in Gram-negative bacteria that can limit the penetration of amphiphilic compounds such as lipopeptides. In addition, the relatively stronger activity of amylimycin A (**1**) suggests that structural variations in the lipid moiety may influence antibacterial potency (Table 3).

In this study, three unreported lipopeptides, amylimycins A–C (**1**–**3**), were isolated from the ethyl acetate extract of *B*. *amyloliquefaciens*, and their structures were characterized. These compounds possess a distinctive structural feature in which the β-amino fatty acid moiety contains two methyl branches along the alkyl chain. Previously reported bacillomycin D analogs typically possess β-amino fatty acid chains that are either straight-chain or contain a single methyl branch. In contrast, amylimycins A–C contain a rare dual-branched β-amino fatty acid moiety [18]. Although *iso*- or *anteiso*-type fatty acid chains are commonly observed in lipopeptides [18], the presence of β-amino fatty acids containing two methyl branches is highly unusual in natural products. To the best of our knowledge, this is only the second reported lipopeptide containing this type of dual-branched β-amino acid, following amylifactins [19]. This unique structural characteristic distinguishes the amylimycins from known bacillomycin D analogs and highlights their structural novelty.

## 3. Experimental Section

### 3.1. General Experimental Procedures

Ultraviolet (UV) spectra were acquired using a Cary 100 UV–VIS spectrophotometer (Varian, Palo Alto, CA, USA) with a 1 cm micro quartz cuvette, and IR spectra were obtained using a Cary 630 Fourier transform IR spectrometer (Agilent Technologies, Santa Clara, CA, USA). Optical rotations were measured using an Optronic P3000 polarimeter (KRÜSS GmbH, Hamburg, Germany). The ^1^H (850 MHz) and ^13^C (212 MHz) NMR experiments were performed using a Bruker 850 MHz NMR spectrometer (Bruker Corp., Billerica, MA, USA) at NCIRF, Seoul, Republic of Korea, while the ^1^H (900 MHz) and ^13^C (225 MHz) NMR experiments were performed using a 900 MHz NMR spectrometer (Bruker Corp.) at the Korea Basic Science Institute, Ochang, Republic of Korea. An Agilent 6530 iFunnel quadrupole-time of flight mass spectrometer (Q-TOF-MS) linked with an Agilent 1290 UHPLC system was used to acquire high-resolution electrospray ionization mass spectrometry (HR-ESI-MS) data. The compounds were purified using an Agilent 1100 series capillary LC system combined with a Waters Micromass ZQ mass spectrometer (Waters Corp., Milford, MA, USA).

### 3.2. Bacterial Isolation

The halophyte *Suaeda maritima* (L.) Dumort was obtained from the tidal flats of Songdo-dong, Incheon, Republic of Korea (37° 23′ 10.7″ N, 126° 40′ 38.9″ E). The whole parts of *S*. *maritima* were disinfected by soaking in 5% NaClO for 5 min and then wiped with 70% aqueous ethanol. The sterilized parts were flaked and placed on solid isolation media for 14 days to isolate endophytes: Czapek-Dox media with sea salt (30 g sucrose, 2 g NaNO_3_, 1 g K_2_HPO_4_, 0.5 g MgCl_2_, 0.5 g KCl, 0.01 g FeCl_2_, 100 mg cycloheximide, 33 g sea salt, and 18 g agar per liter of sterilized water); chitin media with sea salt (6 g chitin, 0.75 g K_2_HPO_4_, 0.5 g MgSO_4_·7H_2_O, 3.5 g KH_2_PO_4_, 10 mg FeSO_4_·7H_2_O, 10 mg MnCl_2_·4H_2_O, 10 mg ZnSO_4_·7H_2_O, 100 mg cycloheximide, 33 g sea salt, and 36 g agar per liter of sterilized water); and A1 media with sea salt (10 g starch, 4 g yeast extract, 2 g peptone, 100 mg cycloheximide, 33 g sea salt, and 18 g agar per liter of sterilized water). To obtain single strains, each colony from the isolation media was inoculated into fresh solid media. The marine-derived strain W2-2 was identified as *B. amyloliquefaciens* based on 16S rRNA gene sequence analysis using two primer pairs: 785F (5′-GGA TTA GAT ACC CTG GTA-3′) and 907R (5′-CCG TCA ATT CMT TTR AGT TT-3′). The sequence for the 16S rRNA of strain W2-2 has been submitted to the NCBI (accession number PQ565561).

### 3.3. Bacterial Cultivation and Purification of Amylimycins A–C

The W2-2 strain was cultured on a solid LB-PDB mixed medium consisting of 5 g tryptone, 2.5 g yeast extract, 5 g NaCl, 2 g potato starch, 10 g dextrose, 33 g sea salt, and 18 g agar per liter of sterilized water at 28 °C. The culture was inoculated into a 250 mL Erlenmeyer flask containing 150 mL of LB-PDB mixed broth and incubated at 28 °C with shaking at 200 rpm for 7 days. Subsequently, 100 mL of seed culture was inoculated into each 2 L Erlenmeyer flask containing 1.2 L of LB-PDB mixed broth. The culture was fermented for 3 days at 28 °C with shaking at 200 rpm. The entire culture (12 L in total) was subjected to extraction using ethyl acetate/water partitioning. Following drying with anhydrous sodium sulfate, the ethyl acetate layer was concentrated *in vacuo*. The dried extracts were loaded onto a prepacked S*Pure SPE-C18 column (SPure Pte Ltd., Singapore) and eluted stepwise using 20%, 40%, 60%, 80%, and 100% aqueous methanol. Amylimycins were eluted in 60% aqueous methanol. The 60% aqueous methanol fraction was further purified via semipreparative LC-MS using 45% aqueous acetonitrile with a 0.1% formic acid solvent system over 30 min (flow rate: 3 mL/min) to yield pure compounds, such as amylimycin A (*R*_t_ 23.1 min), amylimycin B (*R*_t_ 18.6 min), and amylimycin C (*R*_t_ 16.7 min).

Amylimycin A (**1**):

White amorphous powder; IR *v*_max_ (ATR) 3309, 2920, 2831, 1651, and 1024 cm^−1^; UV (MeOH) λ_max_ 202, 220, and 270 nm; HR-ESI-MS *m*/*z* 1073.5885 [M + H]^+^ (calculated for C_51_H_81_N_10_O_15_ *m*/*z* 1073.5877) (Figure S23); ^1^H NMR (DMSO-*d*_6_, 900 MHz); and ^13^C NMR (DMSO-*d*_6_, 225 MHz).

Amylimycin B (**2**):

White amorphous powder; IR *v*_max_ (ATR) 3306, 2924, 2833, 1654, and 1028 cm^−1^; UV (MeOH) λ_max_ 202, 220, and 270 nm; HR-ESI-MS *m*/*z* 1059.5681 [M + H]^+^ (calculated for C_50_H_79_N_10_O_15_ *m*/*z* 1059.5720) (Figure S23); ^1^H NMR (DMSO-*d*_6_, 850 MHz); and ^13^C NMR (DMSO-*d*_6_, 212 MHz).

Amylimycin C (**3**):

White amorphous powder; IR *v*_max_ (ATR) 3307, 2923, 2833, 1652, and 1022 cm^−1^; UV (MeOH) λ_max_ 202, 220, and 270 nm; HR-ESI-MS *m*/*z* 1045.5538 [M + H]^+^ (calculated for C_49_H_77_N_10_O_15_ *m*/*z* 1045.5564) (Figure S23); ^1^H NMR (DMSO-*d*_6_, 850 MHz); and ^13^C NMR (DMSO-*d*_6_, 212 MHz).

### 3.4. Analysis of Metabolites and Molecular Networking

To analyze the metabolites of *B*. *amyloliquefaciens*, molecular networks based on tandem MS were constructed using the Global Natural Product Social Molecular Network (GNPS) platform. The ethyl acetate extract of *B*. *amyloliquefaciens* culture was dried *in vacuo*, dissolved in methanol at a concentration of 250 μg/mL, and analyzed with LC-MS using a YMC-Triart C18 column (150 × 2.0 mm, 5 μm) (YMC Korea, Seongnam, Republic of Korea). The MS experiment was conducted under the following conditions: a drying gas temperature of 300 °C with a flow rate of 8 L/min, a sheath gas temperature of 350 °C with a flow rate of 11 L/min, a capillary voltage of +3.5 kV, and operation in positive ion mode. The MS/MS data of the *B*. *amyloliquefaciens* extract were converted to GNPS-compatible format (mzML) using the MS-Convert program, and the converted files were then used to build an MS/MS molecular network via the GNPS web server: https://gnps.ucsd.edu/ProteoSAFe/static/gnps-splash.jsp (accessed on 27 May 2026). The parameters were set as follows: precursor ion mass tolerance, 2.0 Da; product ion tolerance, 0.05 Da; molecular network cosine score, 0.5; minimum number of matched fragment ions, 6; and minimum cluster size, 2. After the analysis, the data were visualized using Cytoscape 3.10.3 software [20,21].

### 3.5. Determination of the Configuration of Amino Acids in Amylimycins A–C

The absolute configurations of amino acids in amylimycins A–C (**1**–**3**) were determined using the advanced Marfey’s method [22]. Amylimycin A (**1**, 1 mg) was hydrolyzed with 0.5 mL of 6 N aqueous HCl at 115 °C for 24 h under stirring. The reaction vials were then cooled in an ice bath for 3 min. The mixtures were dissolved in water and evaporated under reduced pressure. To completely remove residual HCl, this process was repeated three times, followed by lyophilization for 24 h. The resulting acid hydrolysates containing free amino acids were divided into two portions, each dissolved in 100 μL of 1 N NaHCO_3_. Subsequently, 50 μL of either l-FDAA or d-FDAA (10 mg/mL in acetone) was added to each solution. The reaction mixtures were heated at 80 °C for 3 min and then quenched with 50 μL of 2 N HCl, followed by the addition of 300 μL of 50% aqueous acetonitrile. Finally, 10 μL aliquots of each reaction mixture were analyzed by LC-QTOF-MS using a gradient system (10–40% aqueous acetonitrile containing 0.1% formic acid over 40 min for Asn and Tyr(*Bis*), and 20–60% for the other amino acids; flow rate, 0.5 mL/min) on a YMC-Triart C18 column (150 × 2.0 mm, 5 μm) in negative ion mode.

### 3.6. Antibacterial Activity Assay

The antibacterial activities of amylimycin A (**1**), amylimycin B (**2**), and amylimycin C (**3**) were evaluated independently by MIC assays against Gram-positive bacteria, including *B. subtilis* KCCM 11316 and *S. epidermidis* KCCM 35494, and Gram-negative bacteria, including *E. coli* KCCM 11234 and *P. fluorescens* KCCM 11362. Each strain was inoculated into LB broth supplemented with amylimycins in 48-well plates. The compounds were dissolved in DMSO and tested over a concentration range of 0.25–128 μg/mL using twofold serial dilutions. The plates were then incubated at 28 °C with shaking at 180 rpm for 24 h. Bacterial growth was monitored by measuring the absorbance at 600 nm using an Infinite M200 plate reader (Tecan Group Ltd., Männedorf, Switzerland). Kanamycin and DMSO served as positive and negative controls, respectively. The DMSO control showed no detectable inhibition of bacterial growth, confirming that the observed antibacterial activities were attributable to the tested compounds. The MIC was defined as the lowest concentration that completely inhibited visible growth. All assays were performed in triplicate.

## Figures and Tables

**Table 1:** ^1^H spectroscopic data of amylimycins A–C (**1**–**3**).

Position	1 ^a^				2 ^b^				3 ^b^		
		δ_H_	Mult (J in Hz)			δ_H_	Mult (J in Hz)			δ_H_	Mult (J in Hz)
l-Asn-1				l-Asn-1				l-Asn-1			
1	C			1	C			1	C		
2	CH	4.67	m	2	CH	4.66	m	2	CH	4.65	m
3	CH_2_	2.22	m	3	CH_2_	2.22 *	m	3	CH_2_	2.22 *	m
		2.64	m								
4	C			4	C			4	C		
2-NH		8.32	m	2-NH		8.22	m	2-NH		8.20	m
d-Tyr				d-Tyr				d-Tyr			
5	C			5	C			5	C		
6	CH	4.36	m	6	CH	4.34	m	6	CH	4.34	m
7	CH_2_	2.60	m	7	CH_2_	2.68	m	7	CH_2_	2.64	m
		2.98	m			2.97	m			2.98	m
8	C			8	C			8	C		
9/13	CH	7.00	d (8.0)	9/13	CH	6.99	d (8.0)	9/13	CH	7.00	d (8.0)
10/12	CH	6.62	d (8.0)	10/12	CH	6.61	d (8.0)	10/12	CH	6.61	d (8.0)
11	C			11	C			11	C		
6-NH		7.56	d (8.0)	6-NH		7.59	m	6-NH		7.57	m
d-Asn-2				d-Asn-2				d-Asn-2			
14	C			14	C			14	C		
15	CH	4.50	m	15	CH	4.50	m	15	CH	4.50	m
16	CH_2_	2.31	m	16	CH_2_	2.31	m	16	CH_2_	2.31 *	m
		2.67	dd (5.0, 15.0)			2.62	m				
17	C			17	C			17	C		
15-NH		8.54	d (6.5)	15-NH		8.23	m	15-NH		8.22	m
l-Pro				l-Pro				l-Pro			
18	C			18	C			18	C		
19	CH	4.17	m	19	CH	4.19	m	19	CH	4.20	m
20	CH_2_	2.05 *	m	20	CH_2_	2.02 *	m	20	CH_2_	2.04 *	m
21	CH_2_	1.77 *	m	21	CH_2_	1.77 *	m	21	CH_2_	1.76 *	m
22	CH_2_	3.30	m	22	CH_2_	3.46 *	m	22	CH_2_	3.47 *	m
		3.38	m								
l-Glu				l-Glu				l-Glu			
23	C			23	C			23	C		
24	CH	4.58	dd (5.5, 14.0)	24	CH	4.57	m	24	CH	4.56	m
25	CH_2_	1.87	m	25	CH_2_	1.86	m	25	CH_2_	1.84	m
		1.93	m			1.93	m			1.91	m
26	CH_2_	2.20 *	m	26	CH_2_	2.17 *	m	26	CH_2_	2.18 *	m
27	C			27	C			27	C		
24-NH		8.04	m	24-NH		8.08	m	24-NH		8.06	m
d-Ser				d-Ser				d-Ser			
28	C			28	C			28	C		
29	CH	4.36	m	29	CH	4.36	m	29	CH	4.36	m
30	CH_2_	3.57 *	m	30	CH_2_	3.65 *	m	30	CH_2_	3.65 *	m
29-NH		8.42	d (6.0)	29-NH		8.47	m	29-NH		8.45	m
l-Thr				l-Thr				l-Thr			
31	C			31	C			31	C		
32	CH	4.07	m	32	CH	4.08	m	32	CH	4.09	m
33	CH	4.16	m	33	CH	4.14	m	33	CH	4.14	m
34	CH_3_	1.01	d (6.5)	34	CH_3_	1.00	d (6.5)	34	CH_3_	1.00	d (6.5)
32-NH		8.06	d (9.0)	32-NH		8.06	m	32-NH		8.06	m
β-amino acid				β-amino acid				β-amino acid			
35	C			35	C			35	C		
36	CH_2_	2.19	m	36	CH_2_	2.38 *	dd (5.5, 1.5)	36	CH_2_	2.39 *	dd (5.5, 1.5)
		2.39	m	37	CH	3.88	m	37	CH	3.88	m
37	CH	4.10	m	38	CH_2_	1.50 *	m	38	CH_2_	1.55 *	m
38	CH_2_	1.53 *	m	39–42	CH_2_	1.23 *	m	39–41	CH_2_	1.23 *	m
39–43	CH_2_	1.22 *	m	43	CH_2_	1.12 *	m	42	CH_2_	1.12 *	m
44	CH_2_	1.12 *	m	44	CH	1.49	m	43	CH	1.49	dt (13.5, 6.5)
45	CH	1.49	dt (13.5, 6.5)	45	CH_2_	1.05	m	44	CH_2_	1.06 *	m
46	CH_2_	1.05	m			1.25	m	45	CH	1.25	m
		1.25	m	46	CH	1.28	m	46	CH_2_	1.13 *	m
47	CH	1.28	m	47	CH_2_	1.10 *	m	47	CH_3_	0.82	m
48	CH_2_	1.10 *	m	48	CH_3_	0.82	m	48	CH_3_	0.81	m
49	CH_3_	0.82	m	49	CH_3_	0.81	m	49	CH_3_	0.83	d (6.5)
50	CH_3_	0.81	m	50	CH_3_	0.84	d (6.5)	37-NH		7.50	m
51	CH_3_	0.84	d (6.5)	37-NH		7.52	m				
37-NH		7.58	d (8.0)								

^a^ Acquired at 900 MHz for ^1^H in (DMSO-*d*_6_). ^b^ Acquired at 850 MHz for ^1^H in (DMSO-*d*_6_). * Overlapped signals.

**Table 2:** ^13^C spectroscopic data of amylimycins A–C (**1**–**3**).

Position	1 ^a^			2 ^b^			3 ^b^	
		δ_C_			δ_C_			δ_C_
l-Asn-1			l-Asn-1			l-Asn-1		
1	C	170.3	1	C	170.1	1	C	170.0
2	CH	48.8	2	CH	48.8	2	CH	49.1
3	CH_2_	36.8	3	CH_2_	36.8	3	CH_2_	36.8
4	C	171.4	4	C	171.3	4	C	171.5
d-Tyr			d-Tyr			d-Tyr		
5	C	170.4	5	C	170.9	5	C	170.3
6	CH	56.1	6	CH	56.1	6	CH	56.0
7	CH_2_	35.6	7	CH_2_	35.7	7	CH_2_	35.5
8	C	128.1	8	C	129.1	8	C	129.1
9/13	CH	130.1	9/13	CH	130.1	9/13	CH	130.1
10/12	CH	114.8	10/12	CH	114.7	10/12	CH	114.8
11	C	155.6	11	C	155.6	11	C	155.8
d-Asn-2			d-Asn-2			d-Asn-2		
14	C	169.2	14	C	169.3	14	C	169.2
15	CH	48.1	15	CH	48.1	15	CH	48.2
16	CH_2_	36.2	16	CH_2_	36.2	16	CH_2_	36.2
17	C	172.1	17	C	172.1	17	C	172.2
l-Pro			l-Pro			l-Pro		
18	C	169.0	18	C	169.1	18	C	169.1
19	CH	58.7	19	CH	58.6	19	CH	58.7
20	CH_2_	28.6	20	CH_2_	28.6	20	CH_2_	28.6
21	CH_2_	24.5	21	CH_2_	24.5	21	CH_2_	24.7
22	CH_2_	46.6	22	CH_2_	46.7	22	CH_2_	46.8
l-Glu			l-Glu			l-Glu		
23	C	171.5	23	C	171.3	23	C	171.0
24	CH	49.9	24	CH	49.5	24	CH	50.0
25	CH_2_	28.5	25	CH_2_	28.4	25	CH_2_	28.4
26	CH_2_	31.3	26	CH_2_	31.5	26	CH_2_	31.6
27	C	174.7	27	C	174.2	27	C	174.2
d-Ser			d-Ser			d-Ser		
28	C	173.3	28	C	173.1	28	C	173.2
29	CH	53.0	29	CH	53.1	29	CH	53.1
30	CH_2_	61.3	30	CH_2_	61.3	30	CH_2_	61.3
l-Thr			l-Thr			l-Thr		
31	C	169.6	31	C	169.6	31	C	169.4
32	CH	58.3	32	CH	58.3	32	CH	58.2
33	CH	65.8	33	CH	65.8	33	CH	65.9
34	CH_3_	20.1	34	CH_3_	20.0	34	CH_3_	20.1
β-amino acid			β-amino acid			β-amino acid		
35	C	169.9	35	C	169.9	35	C	170.0
36	CH_2_	42.6	36	CH_2_	42.5	36	CH_2_	42.9
37	CH	45.4	37	CH	45.8	37	CH	46.1
38	CH_2_	31.9	38	CH_2_	32.0	38	CH_2_	31.9
39–43	CH_2_	26.5–29.1	39–42	CH_2_	26.7–29.1	39–41	CH_2_	26.5–28.9
44	CH_2_	38.4	43	CH_2_	38.5	42	CH_2_	38.5
45	CH	27.4	44	CH	27.3	43	CH	27.4
46	CH_2_	36.0	45	CH_2_	35.9	44	CH_2_	36.0
47	CH	33.8	46	CH	33.4	45	CH	32.9
48	CH_2_	29.0	47	CH_2_	29.1	46	CH_2_	29.1
49	CH_3_	11.2	48	CH_3_	11.2	47	CH_3_	11.3
50	CH_3_	19.6	49	CH_3_	19.1	48	CH_3_	19.1
51	CH_3_	22.6	50	CH_3_	22.4	49	CH_3_	22.5

^a^ Acquired at 225 MHz for ^13^C in (DMSO-*d*_6_). ^b^ Acquired at 212 MHz for ^13^C in (DMSO-*d*_6_).

**Table 3:** Antibacterial activity (MIC, μg/mL) of amylimycins A–C (**1**–**3**).

Strains	1	2	3	Kanamycin
*B*. *subtilis* KCCM 11316	16	32	32	2
*S*. *epidermidis* KCCM 35494	32	32	64	0.5
*E. coli* KCCM 11234	64	64	>128	1
*P*. *fluorescens* KCCM 11362	>128	>128	>128	1

## Data Availability

Data are contained in the article or Supplementary Materials.

## Supplementary Materials

The following supporting information can be downloaded at https://www.mdpi.com/article/10.3390/md24060218/s1, Figure S1. ^1^H NMR spectrum (900 MHz) of amylimycin A (**1**) in DMSO-*d*_6_; Figure S2. ^13^C NMR spectrum (225 MHz) of amylimycin A (**1**) in DMSO-*d*_6_; Figure S3. COSY NMR spectrum of amylimycin A (**1**) in DMSO-*d*_6_; Figure S4. ROESY NMR spectrum of amylimycin A (**1**) in DMSO-*d*_6_; Figure S5. TOCSY NMR spectrum of amylimycin A (**1**) in DMSO-*d*_6_; Figure S6. HSQC NMR spectrum of amylimycin A (**1**) in DMSO-*d*_6_; Figure S7. HMBC NMR spectrum of amylimycin A (**1**) in DMSO-*d*_6_; Figure S8. ^1^H NMR spectrum (850 MHz) of amylimycin B (**2**) in DMSO-*d*_6_; Figure S9. ^13^C NMR spectrum (212 MHz) of amylimycin B (**2**) in DMSO-*d*_6_; Figure S10. COSY NMR spectrum of amylimycin B (**2**) in DMSO-*d*_6_; Figure S11. Magnified COSY spectrum of amylimycin B (**2**) in DMSO-*d*_6_; Figure S12. ROESY NMR spectrum of amylimycin B (**2**) in DMSO-*d*_6_; Figure S13. TOCSY NMR spectrum of amylimycin B (**2**) in DMSO-*d*_6_; Figure S14. HSQC NMR spectrum of amylimycin B (**2**) in DMSO-*d*_6_; Figure S15. HMBC NMR spectrum of amylimycin B (**2**) in DMSO-*d*_6_; Figure S16. ^1^H NMR spectrum (850 MHz) of amylimycin C (**3**) in DMSO-*d*_6_; Figure S17. ^13^C NMR spectrum (212 MHz) of amylimycin C (**3**) in DMSO-*d*_6_; Figure S18. COSY NMR spectrum of amylimycin C (**3**) in DMSO-*d*_6_; Figure S19. ROESY NMR spectrum of amylimycin C (**3**) in DMSO-*d*_6_; Figure S20. TOCSY NMR spectrum of amylimycin C (**3**) in DMSO-*d*_6_; Figure S21. HSQC NMR spectrum of amylimycin C (**3**) in DMSO-*d*_6_; Figure S22. HMBC NMR spectrum of amylimycin C (**3**) in DMSO-*d*_6_; Figure S23. Mass spectra of amylimycins A–C (**1**–**3**); Figure S24. Marfey’s analysis of amylimycin A (**1**) and standard amino acids (20–60% aqueous acetonitrile containing 0.1% formic acid over 40 min).; Figure S25. Marfey’s analysis of amylimycin A (**1**) and standard amino acids (10–40% aqueous acetonitrile containing 0.1% formic acid over 40 min).
