# Supplementary material for: Amylimycins A–C, New Bacillomycin D Analogs from Marine-Derived Bacillus amyloliquefaciens
Source: Mar Drugs. 2026 Jun 17;24(6):218. doi: 10.3390/md24060218 (PMC13301712; doi:10.3390/md24060218)

Supporting information for

Amylimycins A–C, New Bacillomycin D Analogs from a Marine-Derived *Bacillus amyloliquefaciens*

Jaeyoun Lee [1], Soohyun Um [2,*], and Seung Hyun Kim [1,*]

[1] College of Pharmacy, Yonsei Institute of Pharmaceutical Sciences, Yonsei University, Incheon 21983, South Korea; jaeyoun1024@yonsei.ac.kr
[2] Department of Forest Products and Biotechnology, Kookmin University, Seoul, 02707, Republic of Korea
* Correspondence: kimsh11@yonsei.ac.kr (S.H.K.), drums@kookmin.ac.kr (S.U.)

Table of Contents

S3 : **Figure S1.** ^{1}H NMR spectrum (900 MHz) of amylimycin A (**1**) in DMSO-d_6.
Figure S2. ^{13}C NMR spectrum (225 MHz) of amylimycin A (**1**) in DMSO-d_6.
S4 : **Figure S3.** COSY NMR spectrum of amylimycin A (**1**) in DMSO-d_6.
Figure S4. ROESY NMR spectrum of amylimycin A (**1**) in DMSO-d_6.
S5 : **Figure S5.** TOCSY NMR spectrum of amylimycin A (**1**) in DMSO-d_6.
Figure S6. HSQC NMR spectrum of amylimycin A (**1**) in DMSO-d_6.
S6 : **Figure S7.** HMBC NMR spectrum of amylimycin A (**1**) in DMSO-d_6.
S7 : **Figure S8.** ^{1}H NMR spectrum (850 MHz) of amylimycin B (**2**) in DMSO-d_6.
Figure S9. ^{13}C NMR spectrum (212 MHz) of amylimycin B (**2**) in DMSO-d_6.
S8 : **Figure S10.** COSY NMR spectrum of amylimycin B (**2**) in DMSO-d_6.
Figure S11. Magnified COSY spectrum of amylimycin B (**2**) in DMSO-d_6.
S9 : **Figure S12.** ROESY NMR spectrum of amylimycin B (**2**) in DMSO-d_6.
Figure S13. TOCSY NMR spectrum of amylimycin B (**2**) in DMSO-d_6.
S10 : **Figure S14.** HSQC NMR spectrum of amylimycin B (**2**) in DMSO-d_6.
Figure S15. HMBC NMR spectrum of amylimycin B (**2**) in DMSO-d_6.
S11 : **Figure S16.** ^{1}H NMR spectrum (850 MHz) of amylimycin C (**3**) in DMSO-d_6.
Figure S17. ^{13}C NMR spectrum (212 MHz) of amylimycin C (**3**) in DMSO-d_6.
S12 : **Figure S18.** COSY NMR spectrum of amylimycin C (**3**) in DMSO-d_6.
Figure S19. ROESY NMR spectrum of amylimycin C (**3**) in DMSO-d_6.
S13 : **Figure S20.** TOCSY NMR spectrum of amylimycin C (**3**) in DMSO-d_6.
Figure S21. HSQC NMR spectrum of amylimycin C (**3**) in DMSO-d_6.
S14 : **Figure S22.** HMBC NMR spectrum of amylimycin C (**3**) in DMSO-d_6.
S15 : **Figure S23.** Mass spectra of amylimycins A–C (**1**–**3**).
S16 : **Figure S24.** Marfey's analysis of amylimycin A (**1**) and standard amino acids (20–60% aqueous acetonitrile containing 0.1% formic acid over 40 min).
S18 : **Figure S25.** Marfey's analysis of amylimycin A (**1**) and standard amino acids (10–40% aqueous acetonitrile containing 0.1% formic acid over 40 min).

Figure S1. ^{1}H NMR spectrum (900 MHz) of amylimycin A (**1**) in DMSO-d_6.

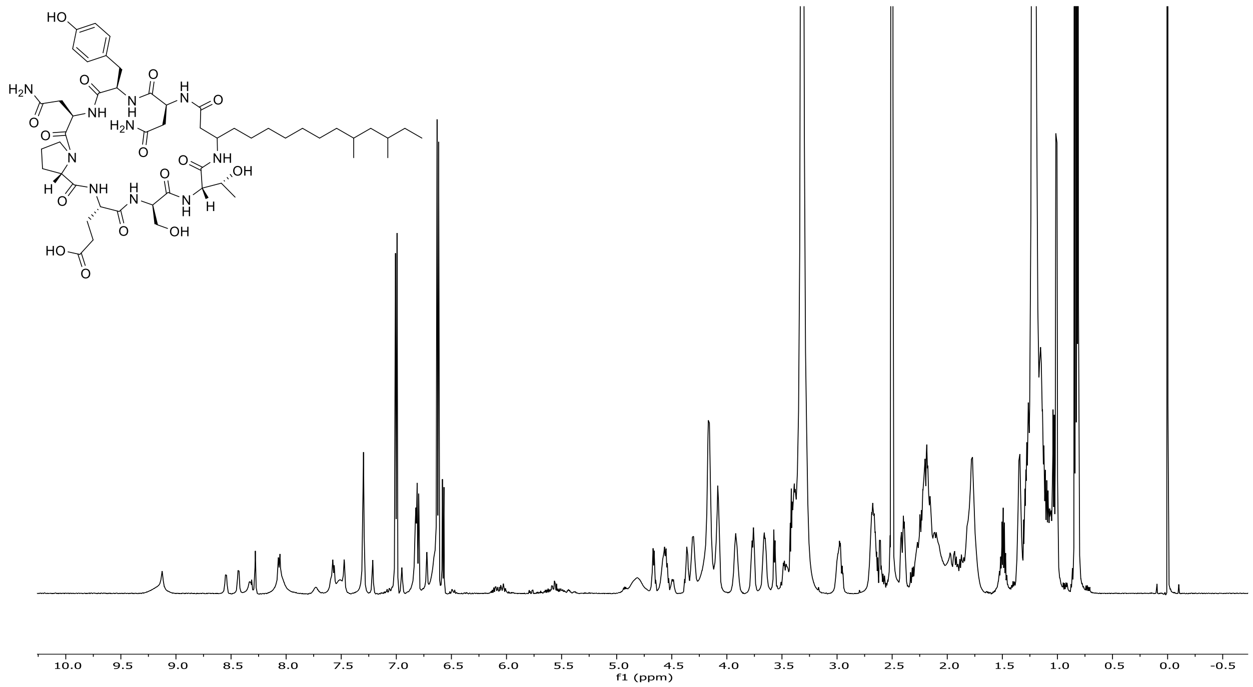

Figure S2. ^{13}C NMR spectrum (225 MHz) of amylimycin A (**1**) in DMSO-d_6.

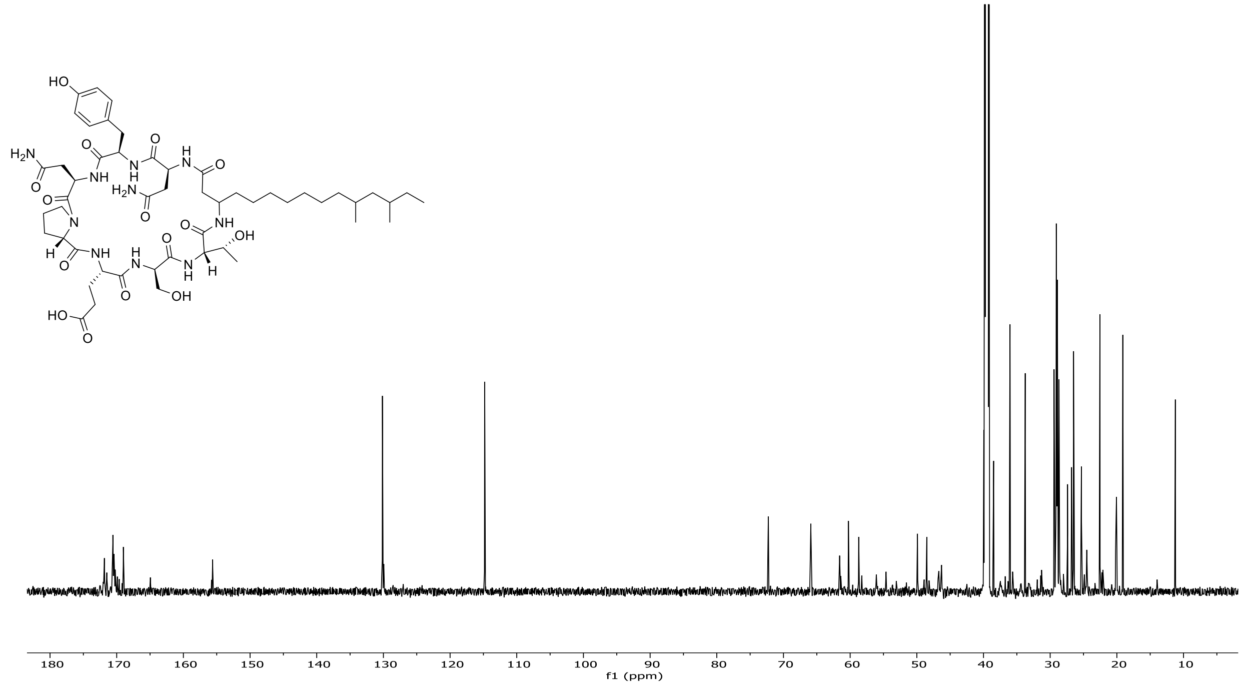

Figure S3. COSY NMR spectrum of amylimycin A (**1**) in DMSO-*d*6.

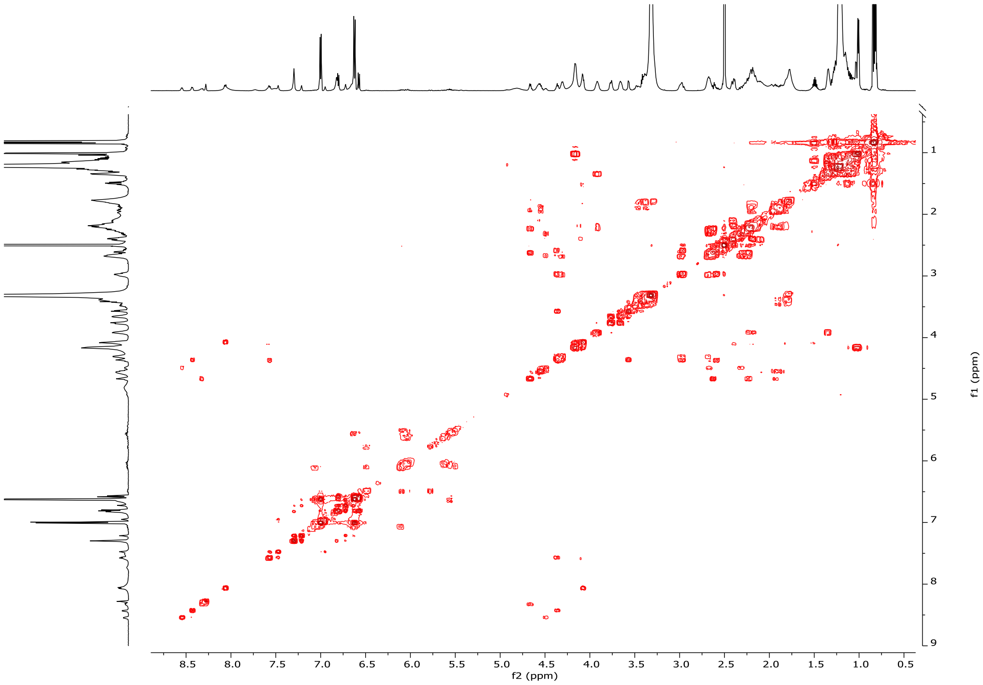

Figure S4. ROESY NMR spectrum of amylimycin A (**1**) in DMSO-*d*6.

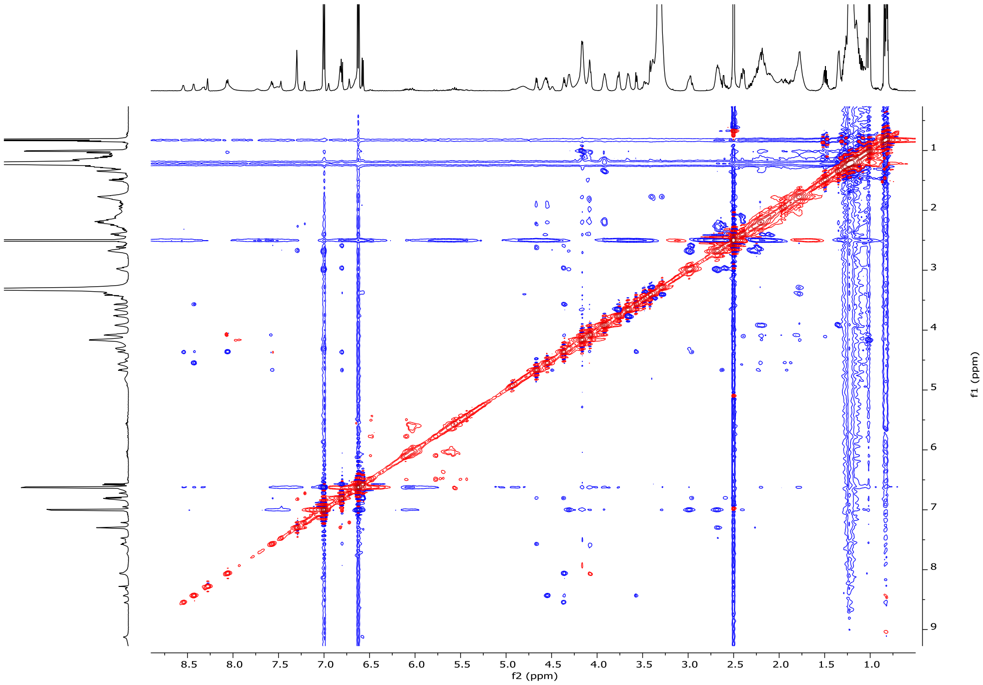

Figure S5. TOCSY NMR spectrum of amylimycin A (**1**) in DMSO-d_6.

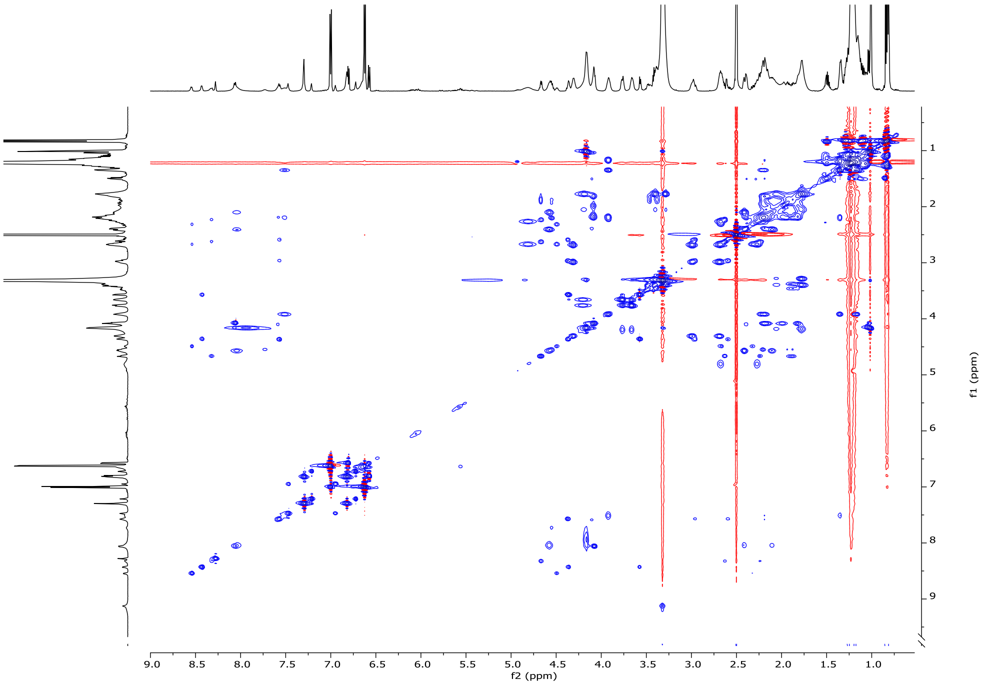

Figure S6. HSQC NMR spectrum of amylimycin A (**1**) in DMSO-d_6.

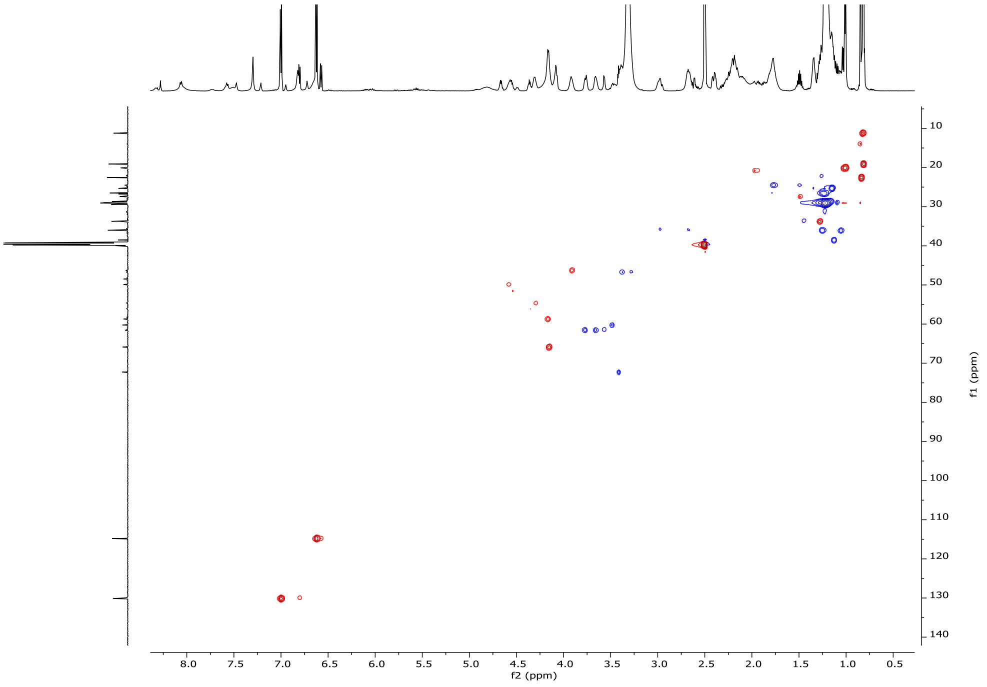

Figure S7. HMBC NMR spectrum of amylimycin A (**1**) in DMSO-d_6.

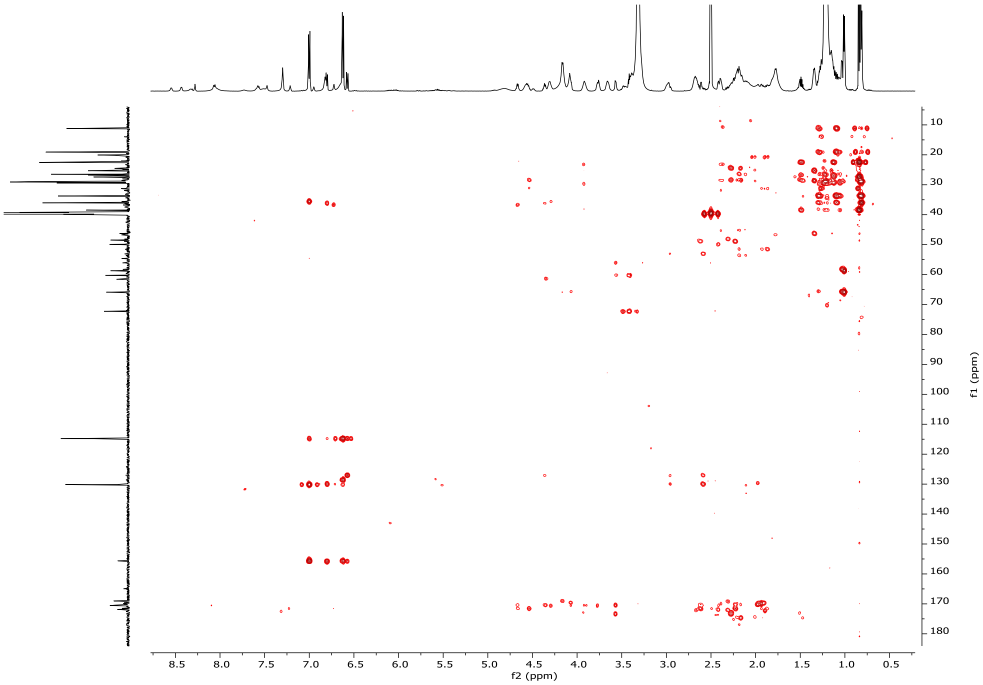

Figure S8. ^{1}H NMR spectrum (850 MHz) of amylimycin B (**2**) in DMSO-*d*$_6$.

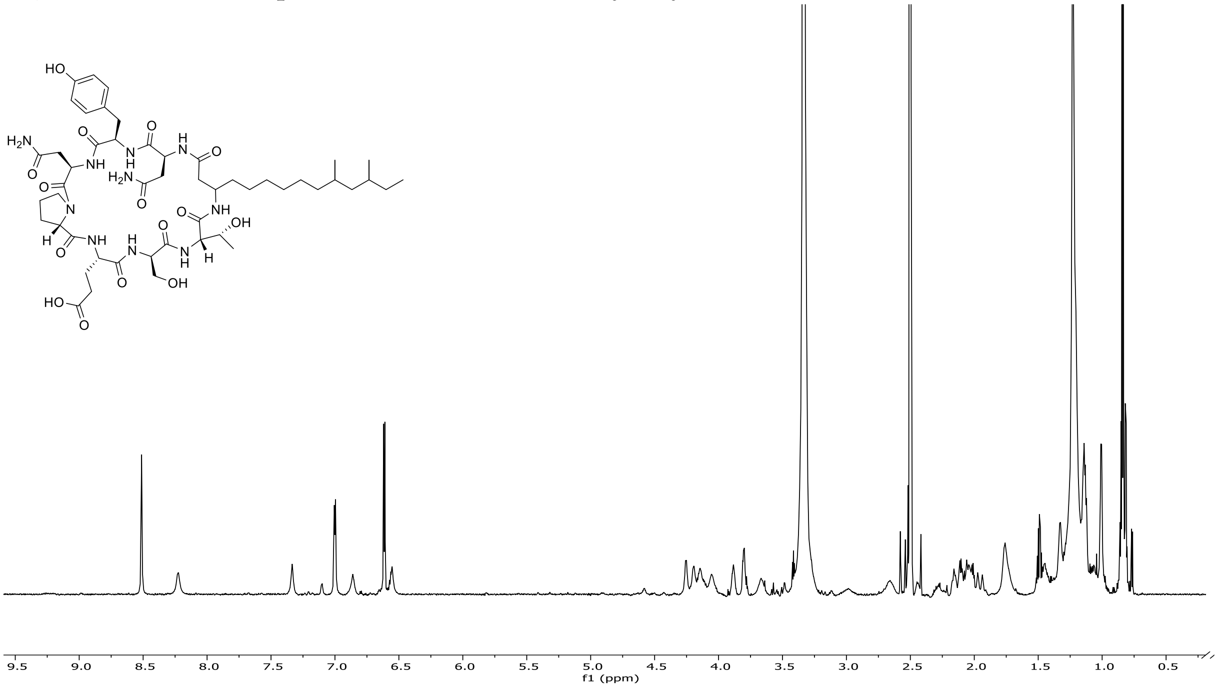

Figure S9. ^{13}C NMR spectrum (212 MHz) of amylimycin B (**2**) in DMSO-*d*$_6$.

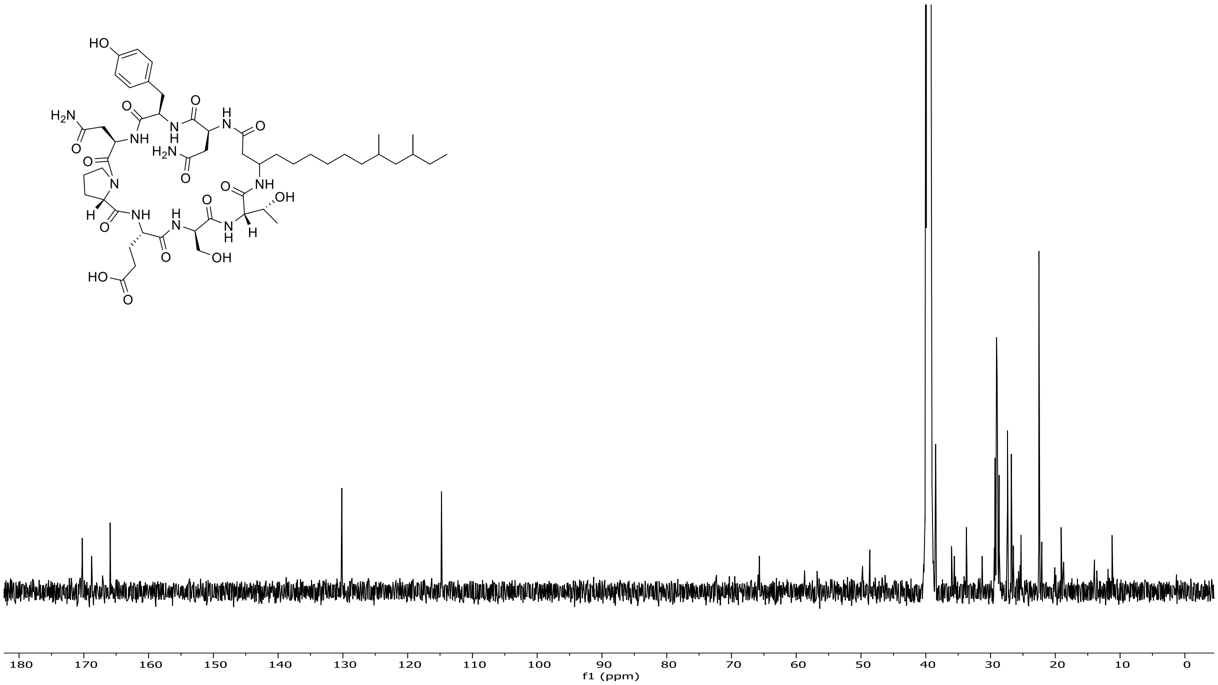

Figure S10. COSY NMR spectrum of amylimycin B (**2**) in DMSO-d_6.

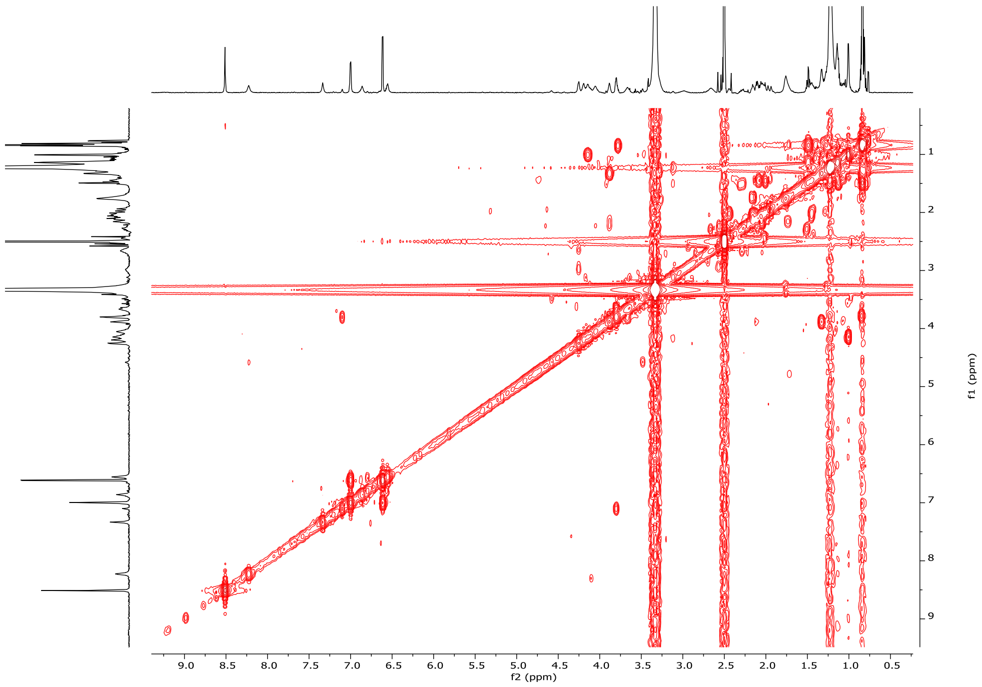

Figure S11. Magnified COSY spectrum of amylimycin B (**2**) in DMSO-d_6.

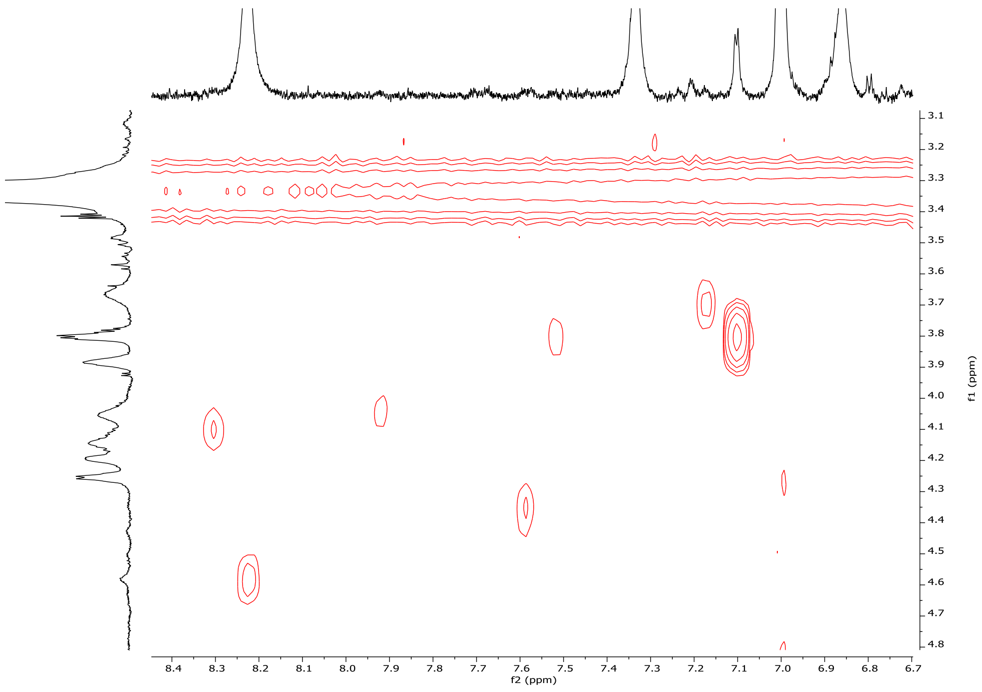

Figure S12. ROESY NMR spectrum of amylimycin B (**2**) in DMSO-*d*6.

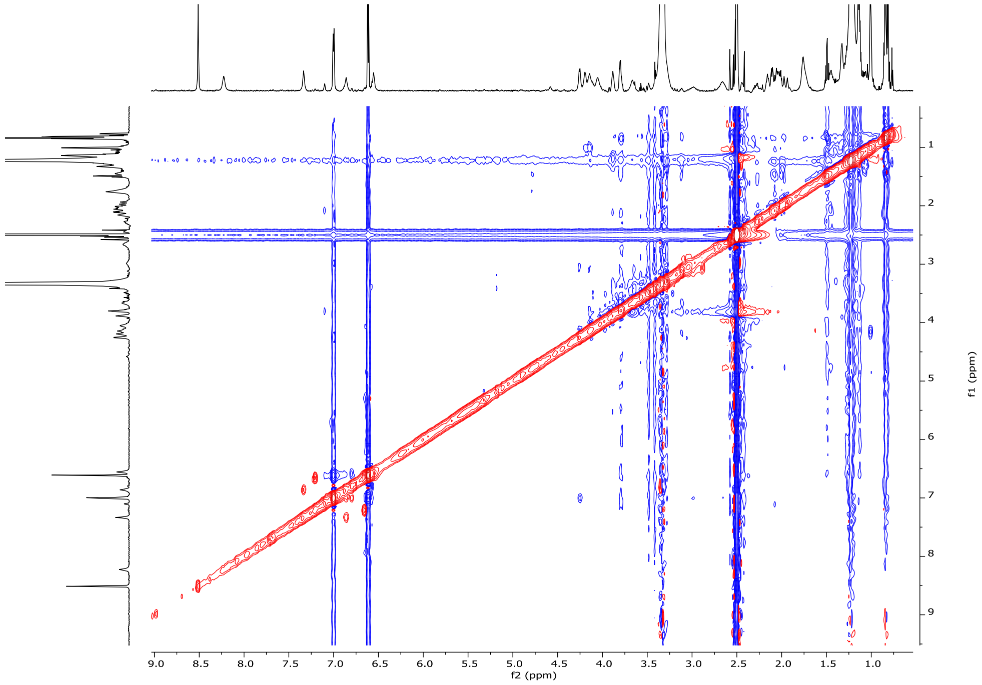

Figure S13. TOCSY NMR spectrum of amylimycin B (**2**) in DMSO-*d*6.

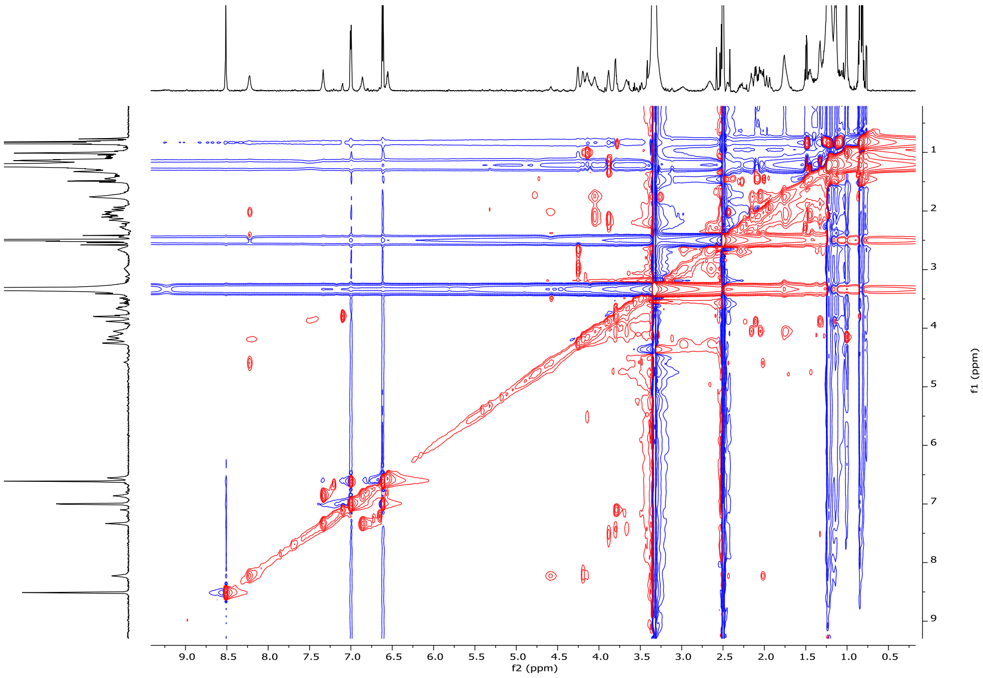

Figure S14. HSQC NMR spectrum of amylimycin B (**2**) in DMSO-d_6.

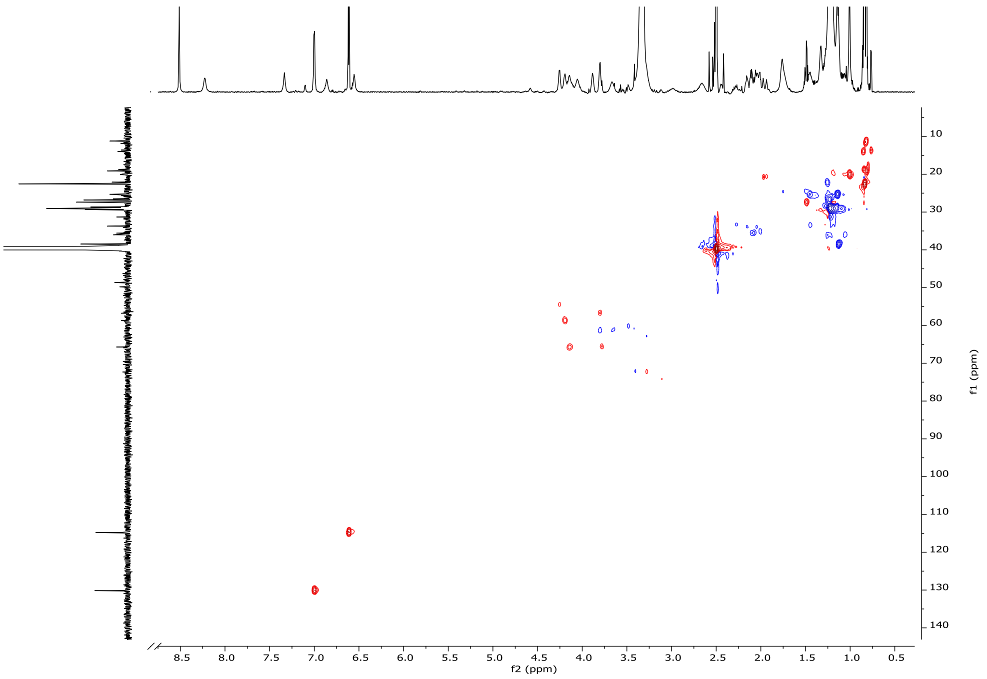

Figure S15. HMBC NMR spectrum of amylimycin B (**2**) in DMSO-d_6.

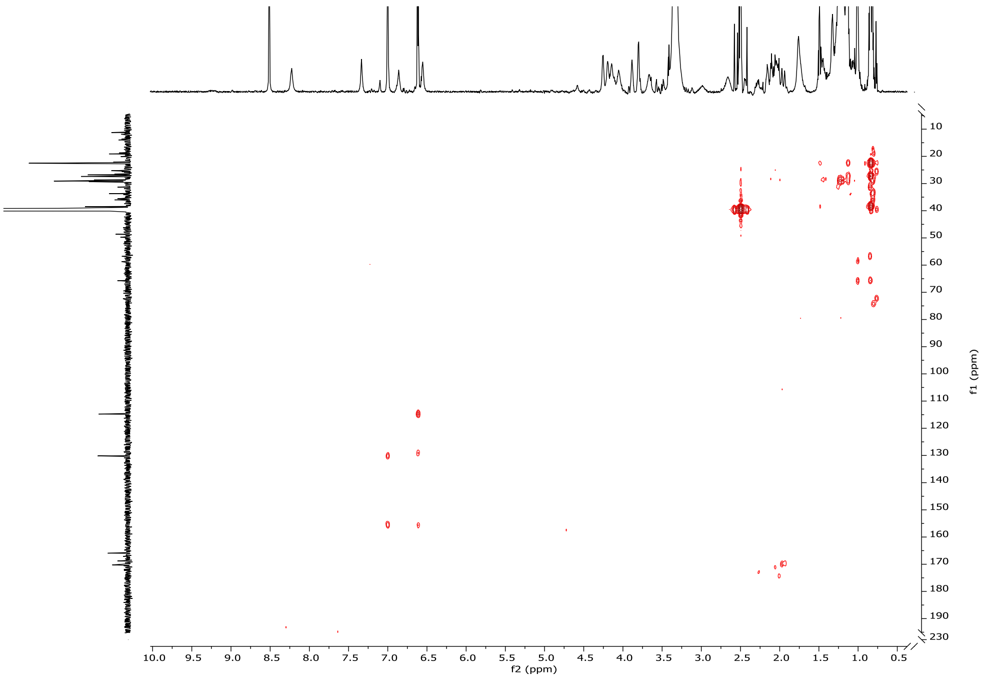

Figure S16. ^{1}H NMR spectrum (850 MHz) of amylimycin C (**3**) in DMSO-d_6.

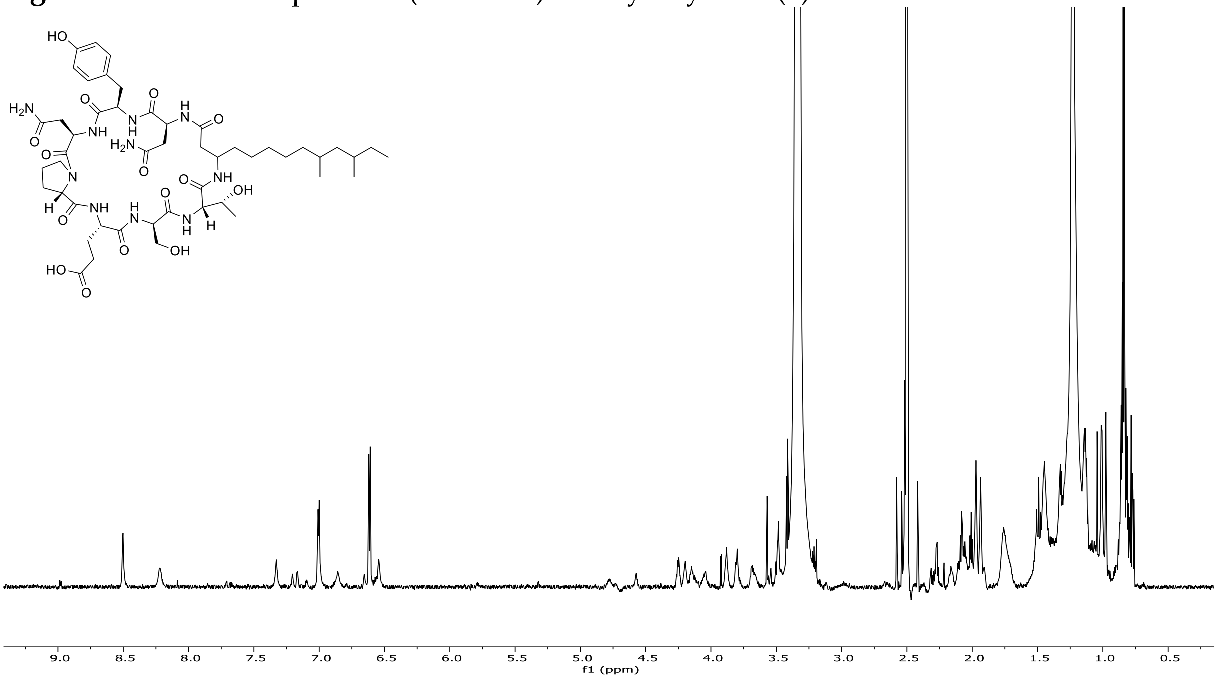

Figure S17. ^{13}C NMR spectrum (212 MHz) of amylimycin C (**3**) in DMSO-d_6.

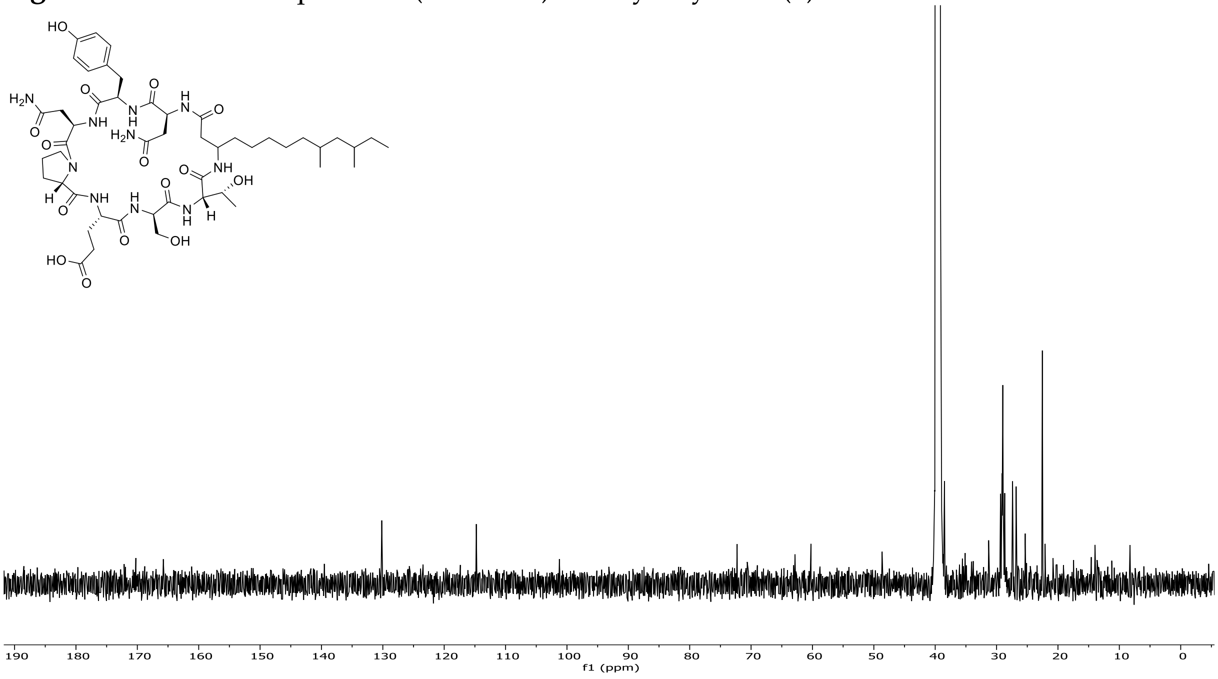

Figure S18. COSY NMR spectrum of amylimycin C (**3**) in DMSO-d_6.

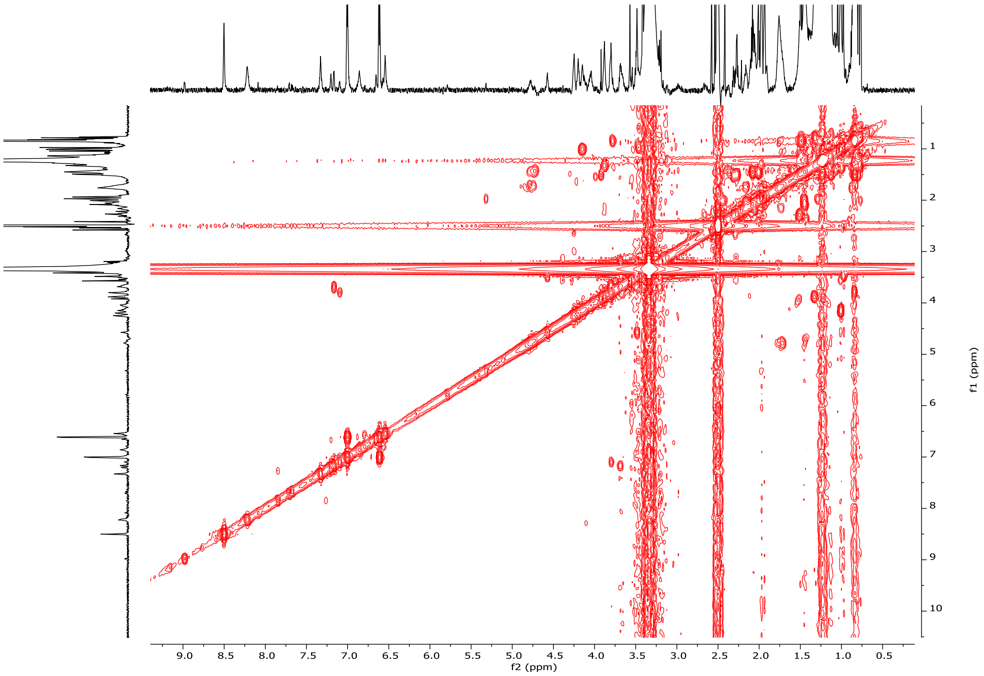

Figure S19. ROESY NMR spectrum of amylimycin C (**3**) in DMSO-d_6.

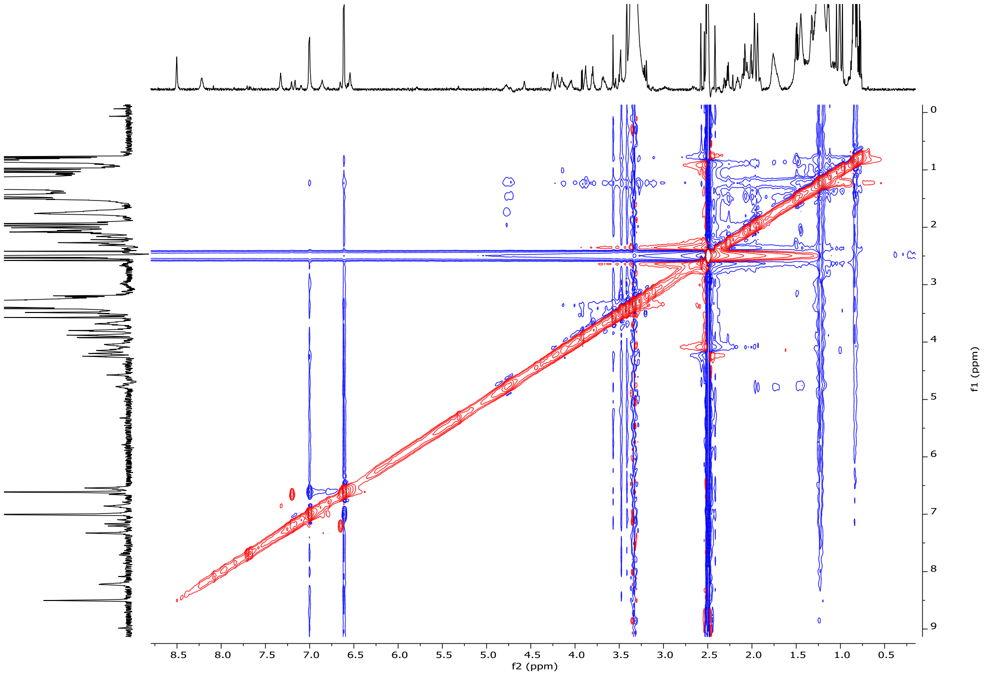

Figure S20. TOCSY NMR spectrum of amylimycin C (**3**) in DMSO-d_6.

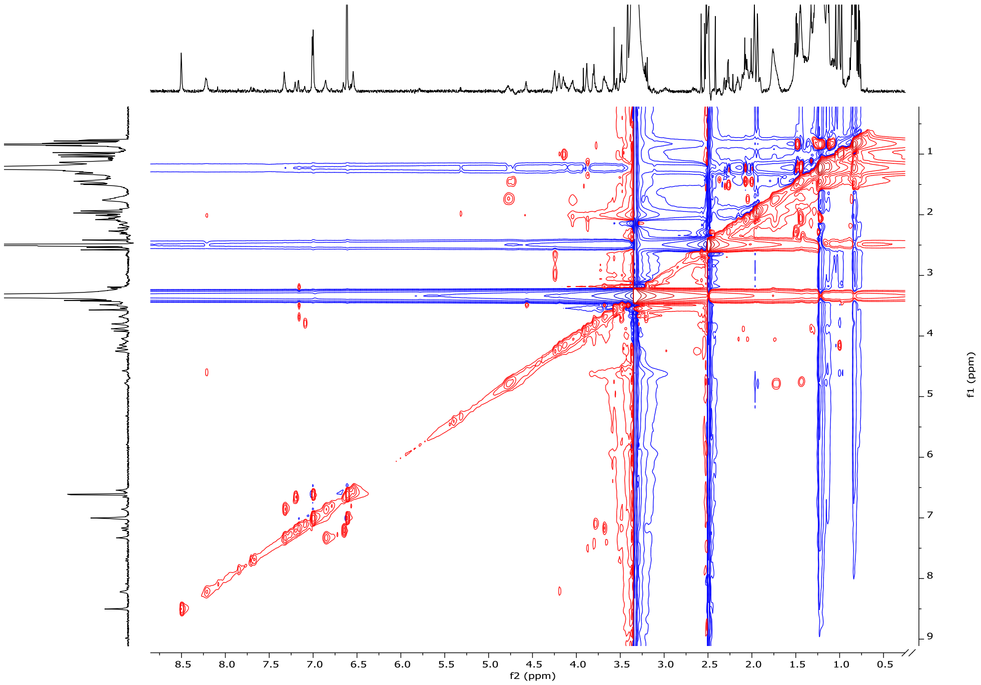

Figure S21. HSQC NMR spectrum of amylimycin C (**3**) in DMSO-d_6.

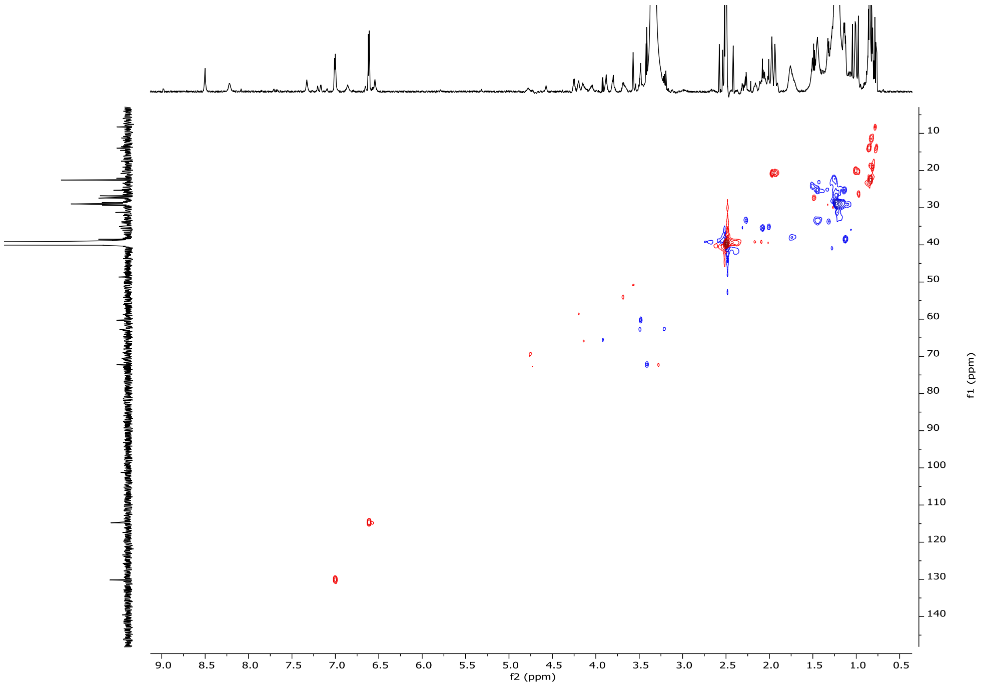

Figure S22. HMBC NMR spectrum of amylimycin C (**3**) in DMSO-d_6.

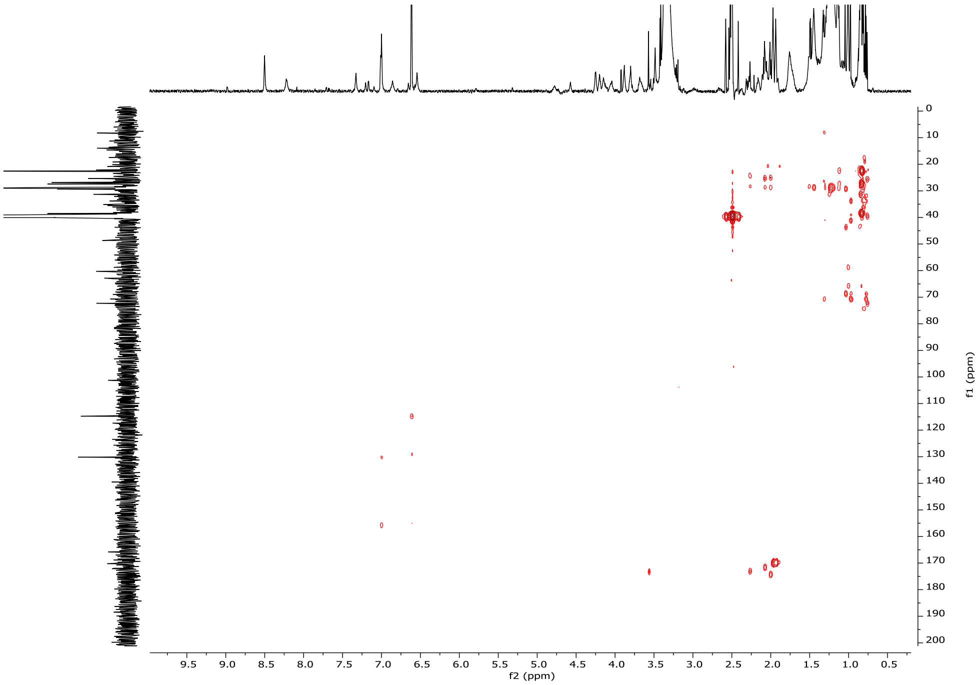

Figure S23. Mass spectra of amylimycins A–C (**1**–**3**).

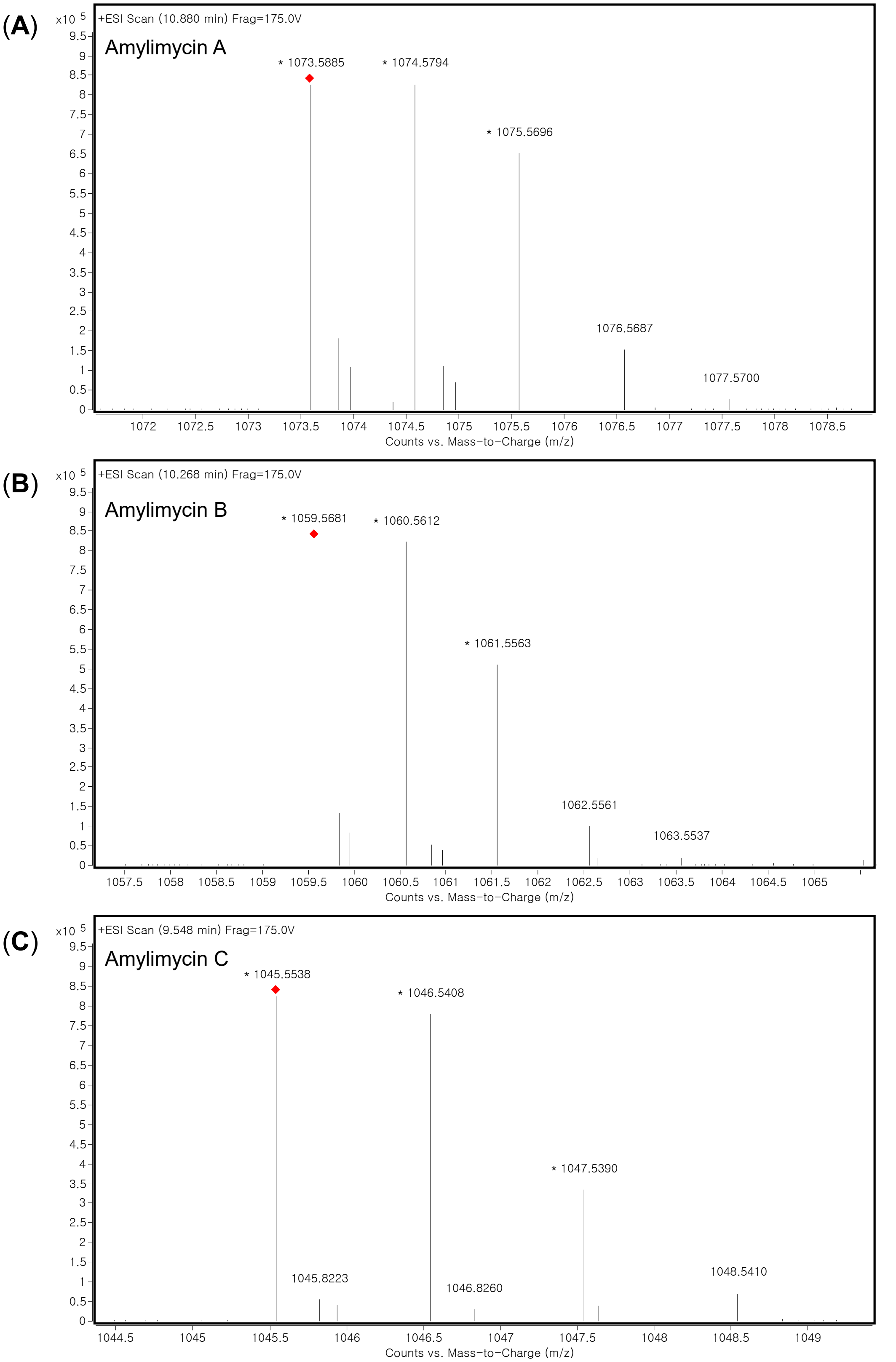

Figure S24. Marfey's analysis of amylimycin A (**1**) and standard amino acids (20–60% aqueous acetonitrile containing 0.1% formic acid over 40 min).

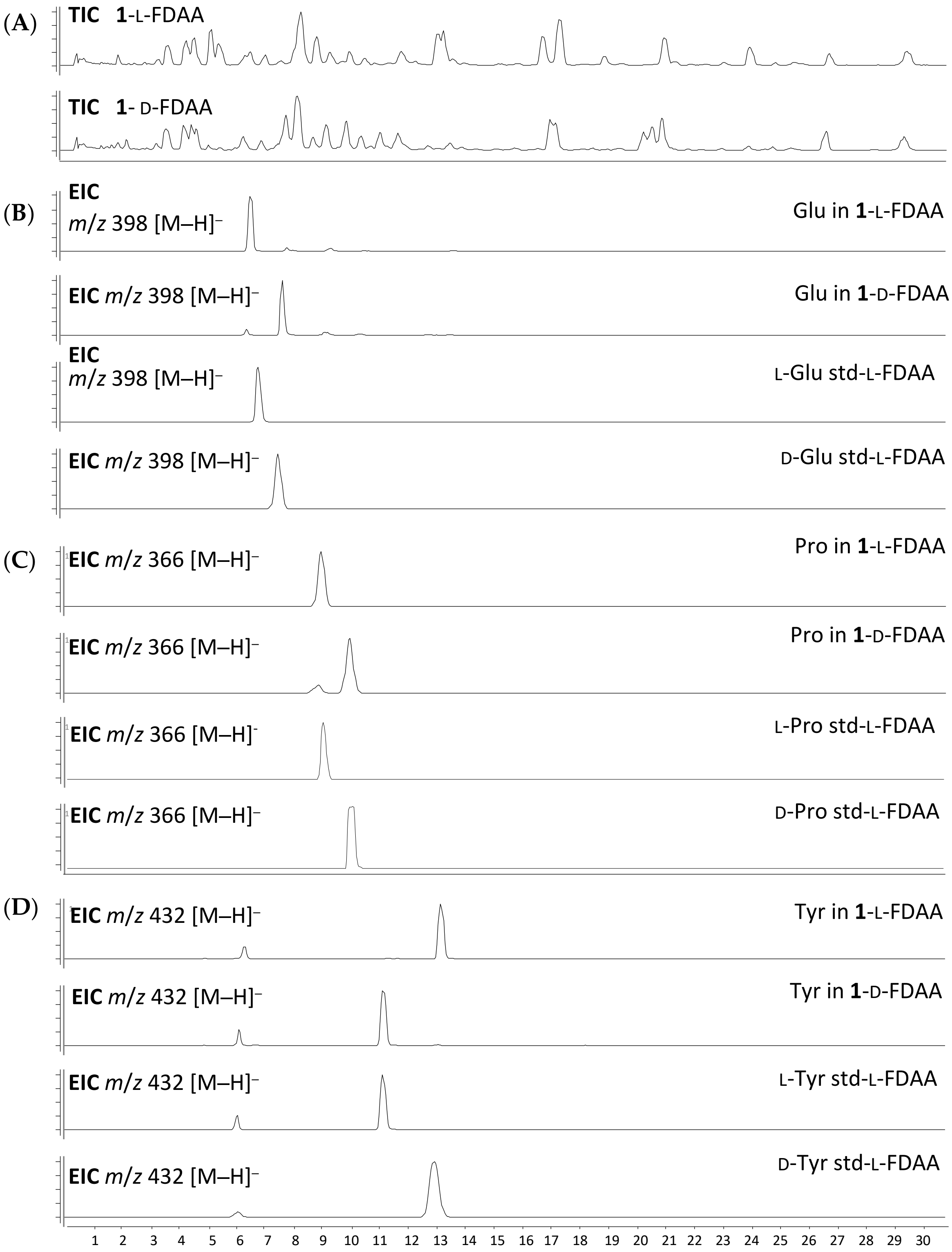

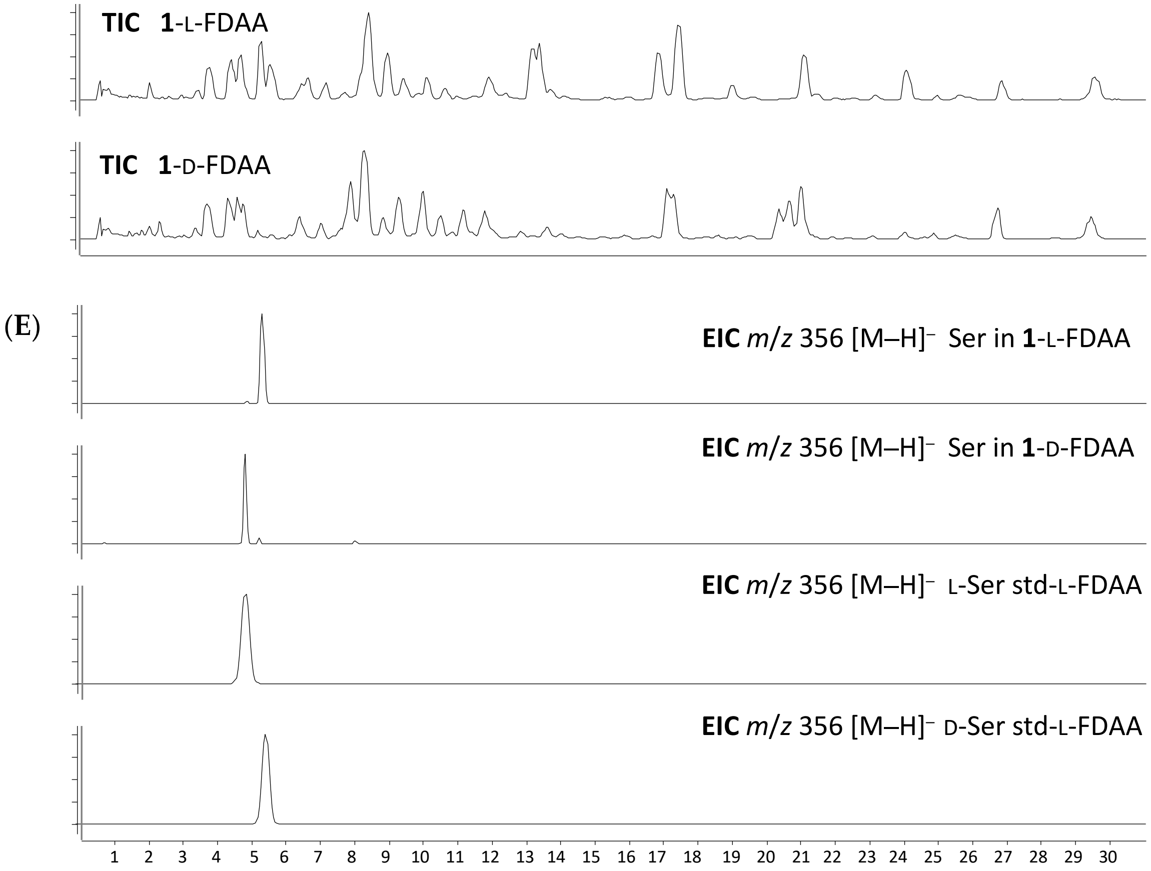
TIC 1-L-FDAA
TIC 1-D-FDAA
(E)
EIC m/z 356 [M–H]⁻ Ser in 1-L-FDAA
EIC m/z 356 [M–H]⁻ Ser in 1-D-FDAA
EIC m/z 356 [M–H]⁻ L-Ser std-L-FDAA
EIC m/z 356 [M–H]⁻ D-Ser std-L-FDAA
1 2 3 4 5 6 7 8 9 10 11 12 13 14 15 16 17 18 19 20 21 22 23 24 25 26 27 28 29 30

Figure S25. Marfey's analysis of amylimycin A (**1**) and standard amino acids (10–40% aqueous acetonitrile containing 0.1% formic acid over 40 min).

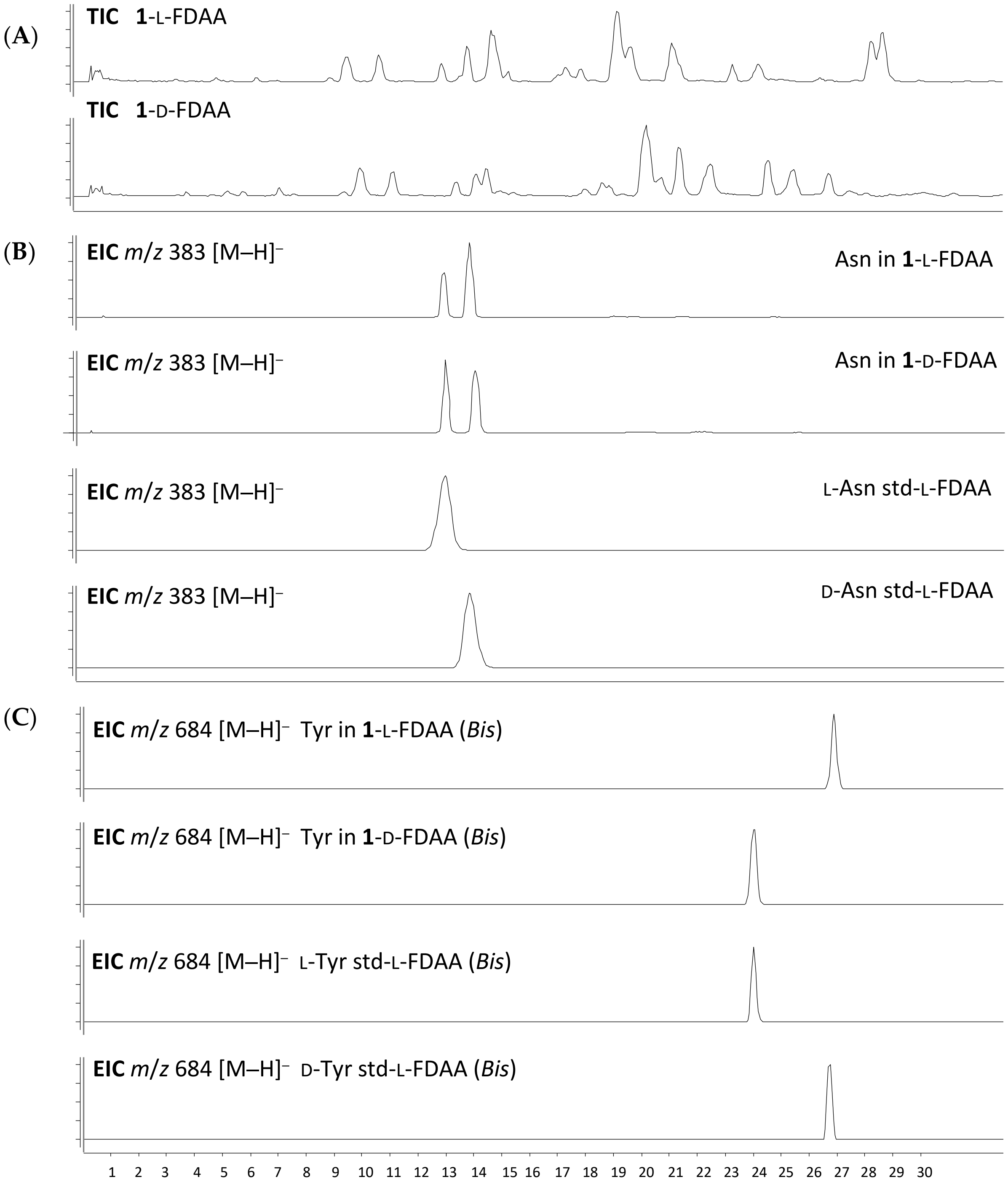

Supplement: Supplementary file 1 [file marinedrugs-24-00218-s001.zip › marinedrugs-4372349-supplementary.pdf]
